# Association of Oral Function and Dysphagia with Frailty and Sarcopenia in Community-Dwelling Older Adults: A Systematic Review and Meta-Analysis

**DOI:** 10.3390/cells11142199

**Published:** 2022-07-14

**Authors:** Kotomi Sakai, Enri Nakayama, Daisuke Yoneoka, Nobuo Sakata, Katsuya Iijima, Tomoki Tanaka, Kuniyoshi Hayashi, Kunihiro Sakuma, Eri Hoshino

**Affiliations:** 1 Comprehensive Unit for Health Economic Evidence Review and Decision Support (CHEERS), Research Organization of Science and Technology, Ritsumeikan University, Kyoto 600-8815, Japan; hoshieri@fc.ritsumei.ac.jp; 2 Heisei Medical Welfare Group Research Institute, Tokyo 151-0053, Japan; sakata.nobuo@hmw.gr.jp; 3 Department of Dysphagia Rehabilitation, Nihon University School of Dentistry, Tokyo 101-8310, Japan; nakayama.en@gmail.com; 4 Infectious Disease Surveillance Center at the National Institute of Infectious Diseases, Tokyo 162-8640, Japan; yoneoka@niid.go.jp; 5 Graduate School of Medicine, The University of Tokyo, Tokyo 113-8654, Japan; 6 Department of Health Policy and Management, School of Medicine, Keio University, Tokyo 160-8582, Japan; 7 Tokyo Foundation for Policy Research, Tokyo 106-6234, Japan; 8 Department of Health Services Research, Faculty of Medicine, University of Tsukuba, Tsukuba 305-8575, Japan; 9 Institute of Gerontology, The University of Tokyo, Tokyo 113-8654, Japan; iijima@iog.u-tokyo.ac.jp (K.I.); tmk-tanaka@iog.u-tokyo.ac.jp (T.T.); 10 Institute for Future Initiatives, The University of Tokyo, Tokyo 113-8654, Japan; 11 Institute of Religion and Culture, Kyoto Women’s University, Kyoto 605-8501, Japan; hayashikuni@kyoto-wu.ac.jp; 12 Institute for Liberal Arts, Environment and Society, Tokyo Institute of Technology, Tokyo 152-8550, Japan; sakuma.k.ac@m.titech.ac.jp

**Keywords:** sarcopenia, frailty, oral function, dysphagia, aging

## Abstract

Studies investigating the associations of oral function and dysphagia with frailty and sarcopenia in community-dwelling older adults are increasing; however, they have not been systematically summarized. We conducted a systematic review to investigate these associations. We searched electronic databases and synthesized relevant data using conventional (frequentist-style) and Bayesian meta-analyses. Twenty-four studies were found to be eligible for our review, including 20 cross-sectional and four prospective cohort studies. Older adults with frailty or sarcopenia had lower tongue pressure, according to the results of conventional meta-analysis (mean difference [95% confidence interval or credible interval]: −6.80 kPa [−10.22 to −3.38] for frailty and −5.40 kPa [−6.62 to −4.17] for sarcopenia) and Bayesian meta-analysis (−6.90 kPa [−9.0 to −4.8] for frailty, −5.35 kPa [−6.78 to −3.89] for sarcopenia). People with frailty had a higher odds ratio (OR) for dysphagia according to the results of conventional meta-analysis (3.99 [2.17 to 7.32]) and Bayesian meta-analysis (1.38 [0.77 to 1.98]). However, the results were inconclusive for people with sarcopenia. A prospective association could not be determined because of the lack of information and the limited number of studies. Decreased oral function and dysphagia can be important characteristics of frailty and sarcopenia in community-dwelling older adults.

## 1. Introduction

The global population is rapidly aging, and older adults are a key population in the medical and healthcare fields. There are many health-related issues in older adults; frailty and sarcopenia are gaining attention because of an increasing trend in their development in the past decade. They are related to adverse events such as falls, hospitalization, disability, and mortality [1,2,3]. Frailty is characterized by increased vulnerability, diminished physiological reserves, and resistance to stressors, with a prevalence of approximately 10.7% in community-dwelling older adults [3,4]. Sarcopenia is characterized by low muscle strength and quantity or quality, with a similar prevalence of approximately 11% in community-dwelling older adults [5,6]. The physical manifestations of these two conditions, such as slow walking speed and weak grip strength, overlap considerably; however, frailty is a broader condition, including limitation in physical activities and exhaustion, than sarcopenia [3]. Managing the symptoms of these two similar conditions is important for maintaining the health of community-dwelling older adults.

Along with increasing studies on physical function, studies on oral function and dysphagia are increasing, as they can also be affected by frailty and sarcopenia. Moreover, ‘oral frailty’ has been defined as a concept similar to that of physical frailty in Japan for indicating the poor oral status of older adults [7]. Recently, articles related to oral frailty were published outside Japan, suggesting increasing global interest in oral and swallowing problems related to frailty and sarcopenia [8,9]. Decreased oral function and dysphagia can lead to malnutrition, aspiration pneumonia, suffocation, and sometimes, death. Thus, the decrease in oral function and development of dysphagia related to frailty and sarcopenia should be prevented by the institution of early intervention in community-dwelling older adults.

Several studies have investigated the association of oral function and dysphagia with frailty and sarcopenia in community-dwelling older adults. However, no systematic review has summarized these associations, especially those focusing on oral function and dysphagia. Therefore, a systematic review of all available data is essential to understand the current situation of these associations, improve community health practice, and conduct effective interventions and useful research on community-dwelling older adults. This systematic review aimed to investigate the association of oral function and dysphagia with frailty and sarcopenia in community-dwelling older adults.

## 2. Materials and Methods

We followed the Meta-analysis of Observational Studies in Epidemiology (MOOSE) guidelines for reporting this systematic review (Table S1) [10]. The review protocol was registered in PROSPERO (CRD42019141277).

### 2.1. Search Strategy

We conducted a systematic search on PubMed, EMBASE, Ichu-Shi Web, ClinicalTrials.gov, and the World Health Organization International Clinical Trials Platform Search Portal from inception to 1 February 2022. We manually searched the bibliographies of the included studies and asked experts in the respective fields to identify missing studies. Specific search strategies were developed by librarians (Table S2). Two reviewers (KS and EN) screened the titles, abstracts, and full texts independently and in duplicate to find studies that met the inclusion criteria, with disagreements resolved by discussion or consulting the third reviewer (EH).

### 2.2. Selection Criteria

We included studies performed in community-dwelling older adults with a mean age of ≥60 years and having frailty and/or sarcopenia according to any definition; studies published in English or Japanese with a prospective or retrospective cohort or case-control or cross-sectional study design were included. Studies including the following participants were excluded: institutionalized older adults such as those in nursing homes and hospitals; more than half of the participants having a history of stroke, head and neck cancer, Parkinson’s disease, or other neuromuscular diseases; only patients with diseases such as diabetes mellitus; or participants who were only outpatients. We excluded gray literature and conference abstracts.

### 2.3. Types of Outcome Measures

The primary outcomes were the following two associations of oral function and dysphagia with frailty and sarcopenia: (1) cross-sectional associations such as whether older adults with frailty or sarcopenia had decreased oral function or dysphagia and (2) longitudinal associations of oral function and dysphagia with frailty and sarcopenia. The secondary outcomes were the association of the presence of decreased oral function and dysphagia at baseline with the adverse outcomes of physical dependence, malnutrition, aspiration pneumonia, suffocation, and death at follow-up. We assessed oral function by evaluating tongue strength and movement, lip strength and movement, suprahyoid muscle strength, and occlusal force, while the presence of dysphagia was determined using any definition proposed by the study authors. We focused on muscle strength-related functions that can be affected by frailty or sarcopenia and not on the chewing ability that can be affected by the condition of the teeth.

### 2.4. Data Extraction

Two reviewers (KS and EN), independently and in duplicate, extracted data from studies that met the inclusion criteria using a prespecified form designed using the data extraction for complicated meta-analysis (DECiMAL) guide [11], and disagreements were resolved by discussion or consulting the third reviewer (EH). The additional data required for meta-analyses were asked for by sending emails to authors of the included studies. In studies that provided some definitions of frailty, we extracted data using the criteria of the Cardiovascular Health Study [3]. Further, we extracted the data pertaining to the frailty and non-frailty groups (individuals who were robust and who had pre-frailty) as well as the pre-frailty and robust groups.

### 2.5. Quality Assessment and Certainty of Evidence

The reviewers (KS and EN) assessed the quality of the included studies using the National Institutes of Health Quality Assessment Tool for Observational Cohort and Cross-Sectional Studies independently, resolving any differences through discussion or by consulting the third reviewer (EH) [12]. The Grading of Recommendations, Assessment, Development, and Evaluation (GRADE) approach was used to rate the certainty of a body of evidence for associations in prospective cohort studies [13]. As the GRADE approach is used for assessing interventions, prognosis, and diagnostic tests, we did not rate the certainty of evidence for outcomes in cross-sectional studies using this approach. The allotted grade was independently rated and discussed by two authors (KS and EN), and when needed, the third reviewer (EH) joined the discussion.

### 2.6. Data Synthesis

We conducted conventional (frequentist-style) meta-analysis (CM) and Bayesian meta-analyses (BM) when studies were adequately similar for synthesis. BM allows the modeling of all parameters for uncertainty. We decided to include both CM and BM for robust results. The medians and interquartile ranges (IQRs) reported by studies conducted with >100 participants per group were converted to means and standard deviations (SDs) using the method described in the Cochrane handbook for meta-analyses [14]. We used the DerSimonian and Laird random-effects method, considering the variations in the included studies for CM. Mean difference (MD) was used when oral function and dysphagia were assessed using the same assessment tool; the standard mean difference (SMD) was used when studies used different assessment tools for continuous variables and odds ratio (OR) for dichotomous variables. To assess the degree of heterogeneity between studies used for CM, we visually inspected the forest plots and estimated the I^2^ statistics; ≥75% of I^2^ statistics was deemed considerable [15]. When the heterogeneity was considerable, we attempted to identify the source of heterogeneity. We conducted BM for outcomes that were reported by at least two studies. The ORs were transformed into logarithmic ORs and standard errors. The two-level hierarchy model was used for assessing trial heterogeneity, which corresponds to the random-effects model in CM. Regarding the prior mean parameter in BM, we assumed a non-informative normal distribution with a mean of 0 and an SD of 100,000 for continuous outcomes and a mean of 0 and an SD of 4 for binary outcomes. Regarding the prior for the heterogeneity parameter τ, we assumed a half-normal distribution with a scale of 0.5 for BM. We planned to conduct analyses separately for sarcopenia and frailty and did not plan any subgroup analyses. For outcomes with more than 10 studies, we planned to explore possible publication biases [16]. All of the analyses were performed using R version 4.1.2. with ‘Meta’ and ‘Bayesmeta’ packages [17,18].

## 3. Results

We identified 710 potentially eligible studies through initial searches, screened 584 titles and abstracts after removing duplicates, and reviewed the full texts of 51 reports. Finally, we included 24 studies and 24 reports with 17,634 participants, including 4549 with pre-frailty, 1327 with frailty, and 629 with sarcopenia in cross-sectional studies. We did not find any ongoing or planned studies related to our review in the trial registers. A PRISMA flow diagram of the study selection process is shown in Figure S1. A list of excluded studies is shown in Table S3. The characteristics of the included studies are summarized in Table 1. Of the included studies, 20 were cross-sectional, and four were prospective cohort studies. A total of 15 studies investigated the association of oral function and dysphagia with frailty, six studies investigated the association of oral function and dysphagia with sarcopenia, and three studies investigated the association of oral function and dysphagia with both frailty and sarcopenia. Eighteen studies were conducted in Japan, while the other studies were conducted in the following countries: Korea, one; Taiwan, two; U.S., one; Spain, one; and Israel, one. Table S4 shows the inclusion and exclusion criteria of the included studies. Table 2 shows the items assessed in the included studies, with the assessment tools used for frailty, sarcopenia, and dysphagia. All of the studies used validated assessment tools for frailty and sarcopenia. Tongue pressure was assessed in 7 of 18 studies, including participants with frailty, and in seven of nine studies, including participants with sarcopenia. The occlusal force was assessed in 9 of 18 studies, including participants with frailty, and in four of nine studies, including participants with sarcopenia. In one study, the association of suprahyoid muscle strength with sarcopenia was assessed using the jaw-opening force as a surrogate index. Oral diadochokinesis (ODK), in the form of tongue and lip motor function, was assessed in 6 of 18 studies, including participants with frailty, and in five of nine studies, including participants with sarcopenia. Dysphagia was assessed in 11 of 18 studies, including participants with frailty, and in seven of nine studies, including participants with sarcopenia. Of the 15 studies that included an assessment for dysphagia, 11 studies included self-reported assessments, eight of which used the Eating Assessment Tool-10 (EAT-10). None of the studies assessed lip strength. Regarding longitudinal associations, the associations of oral function and/or dysphagia at baseline with the presence of frailty, sarcopenia, and/or disability at follow-up were investigated in four prospective cohort studies. We did not find any studies that investigated the association of frailty, sarcopenia, and disability at baseline with oral function and dysphagia at follow-up or the association of oral function and dysphagia at baseline with the outcomes of malnutrition, aspiration pneumonia, suffocation, or death.

### 3.1. Tongue Pressure in Frailty

**Figure 1:**
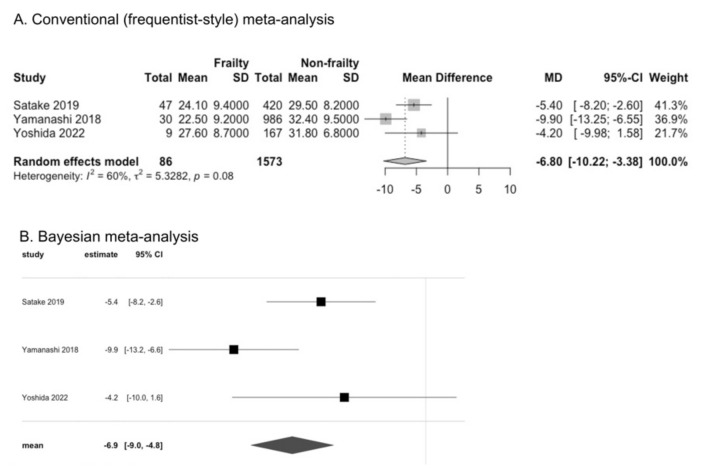
Comparison of tongue pressure between individuals with and without frailty [19,20,21].

We included all five cross-sectional studies that assessed frailty and tongue pressure in the meta-analyses; of these, three studies reported the value of tongue pressure [19,20,21], and two studies categorized tongue pressure into low or normal [22,23]. Shimazaki et al. reported the median tongue pressure; however, we did not include their study in the meta-analyses for the value of tongue pressure because we could not obtain the mean value [23]. This study showed that tongue pressure did not differ significantly among individuals with frailty, pre-frailty, and robustness, as assessed using the Kihon Checklist. All of the studies reported tongue pressure for men and women together. Tongue pressure was significantly lower in individuals with frailty than in those without frailty, according to the results of a conventional (frequentist-style) meta-analysis (CM) (MD −6.80 kPa, 95% confidence interval [CI] −10.24 to −3.38, I^2^ = 60%; three studies). Similarly, the results of the Bayesian meta-analyses (BM) showed that tongue pressure was lower (MD −6.9 kPa, 95% credible interval [CrI] −9.0 to −4.8) in individuals with frailty than in those without frailty (Figure 1). The OR for low tongue pressure, which was defined as tongue pressure < 30 kPa, was higher in individuals with frailty than in those without frailty (OR 1.56, 95% CI 1.08 to 2.26, I^2^ = 2%; two studies), according to the results of CM. However, the results of BM were inconclusive (OR 0.46, 95% CrI −0.23 to 1.19) (Figure S2). Tongue pressure did not differ significantly between individuals with pre-frailty and robust individuals, according to the results of CM (MD −2.64 kPa, 95% CI −6.17 to 0.89, I^2^ = 94%; two studies). However, the results of BM showed that individuals with pre-frailty had lower tongue pressure than robust individuals (MD −2.88 kPa, 95% CrI −4.43 to −1.20) (Figure S3).

### 3.2. Tongue Pressure in Sarcopenia

**Figure 2:**
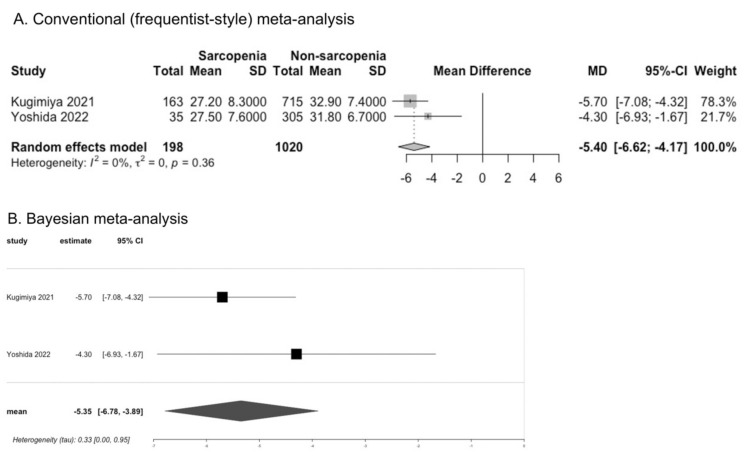
Comparison of tongue pressure between individuals with and without sarcopenia [20,25].

Four studies reported tongue pressure for men and women together, of which three studies reported the value of tongue pressure [20,24,25], and one study categorized tongue pressure (<30 kPa or not) [22]. Kugimiya et al. reported tongue pressure as a median value with an interquartile range (IQR) [25]; we converted this value into a mean value with SD for meta-analysis. Two cross-sectional studies reported tongue pressure according to sex, of which one study (Suzuki et al.) only included women [26,27]. All of the studies assessed tongue pressure in the front of the tongue, except for one study (Chen et al., 2020) that assessed tongue pressure in the middle part of the tongue [24]. The pooled data of the studies that reported tongue pressure for men and women together showed that individuals with sarcopenia had lower tongue pressure in the front part of the tongue, according to the results of CM (MD −5.40 kPa, 95%CI −6.62 to −4.17, I^2^ = 0%; two studies). The results of BM also indicated that tongue pressure was lower in individuals with sarcopenia than in those without sarcopenia (MD −5.35, 95% CrI −6.78 to −3.89) (Figure 2). Women with sarcopenia had lower tongue pressure according to the results of both CM (MD −7.59 kPa, 95%CI −11.89 to −3.28, I^2^ = 78%; two studies) and BM (MD −7.2, 95% CrI −9.3 to −5.1) (Figure S4). Machida et al. reported tongue pressure in men, which was lower in those with sarcopenia than in those without sarcopenia (MD −8.00 kPa, 95%CI −10.39 to −5.61) [26]. Chen et al. reported no significant difference in the middle part of tongue pressure between individuals with sarcopenia and those without sarcopenia (MD −1.60 kPa, 95% CI −6.71 to 3.51) [24]. Nakamura et al. categorized tongue pressure and reported that individuals with sarcopenia had a significantly higher OR for low tongue pressure (<30 kPa) (OR 2.18, 95% CI 1.49 to 3.19) [22].

### 3.3. Occlusal Force in Frailty and Sarcopenia

**Figure 3:**
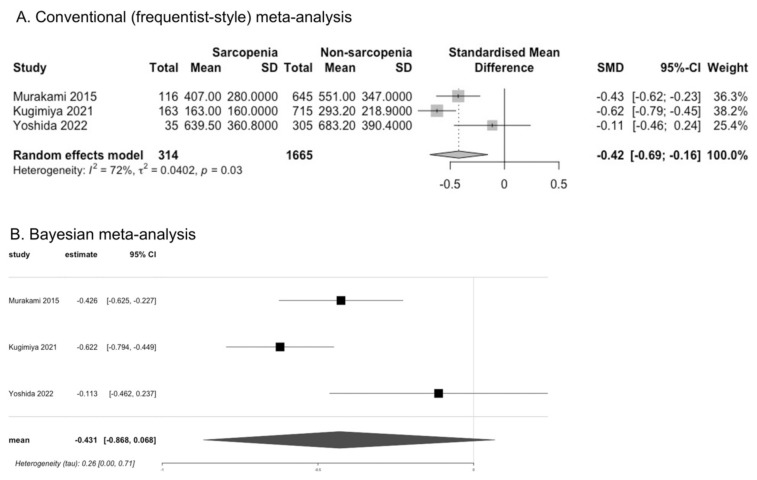
Comparison of occlusal force between individuals with and without sarcopenia [20,25,31].

The occlusal force was assessed in individuals with frailty in one study (Watanabe et al., 2017) according to sex [28]. The occlusal force was significantly lower in men (MD −227.90 N, 95%CI −277.53 to −178.27) and in women (MD −192.00 N, 95% CI −227.53 to −156.47) with frailty than in robust individuals [28]. Kera et al. categorized individuals with frailty into five groups and showed that those with frailty in one group had lower occlusal force than those without frailty for men and women together (MD −220.30 N, 95%CI −287.32 to −153.28) [29]. Horibe et al. showed that individuals with frailty had higher OR for low occlusal force, which was defined as <192.7 N, than those without frailty (OR 2.02, 95% CI 1.04 to 3.91) [30]. For pre-frailty, Watanabe et al. showed significantly lower occlusal force in men (MD −79.10 N, 95% CI −111.88 to −46.32) and women (MD −63.8 N, 95% CI −88.64 to −38.96) with pre-frailty than robust individuals [28]. Three studies assessed occlusal force in individuals with sarcopenia and reported that for men and women together [20,25,31]. We converted the median value with IQR of occlusal force reported in one study (Kugimiya et al.) into the mean value with SD [25]. Individuals with sarcopenia had lower occlusal force than those without sarcopenia, according to the results of CM (SMD: −0.42, 95% CI −0.69 to −0.16, I^2^ = 72%; three studies). However, the results of BM were inconclusive (SMD −0.43, 95% CrI −0.87 to 0.07) (Figure 3). Murakami et al. assessed occlusal force according to sex and showed that individuals with sarcopenia had significantly lower occlusal force than those without sarcopenia in both men (MD −242.00 N, 95% CI −335.77 to −148.23) and women (MD −78.00 N, 95% CI −150.21 to −5.79) [31].

### 3.4. Suprahyoid Muscle Strength in Frailty and Sarcopenia

Machida et al. showed a difference in suprahyoid muscle strength assessed using jaw-opening force as a surrogate index between individuals with and without sarcopenia [26]. Individuals with sarcopenia had lower suprahyoid muscle strength than those without sarcopenia in the study (MD −1.40, 95% CI −1.90 to −0.90). We did not find any studies that investigated suprahyoid muscle strength in individuals with frailty.

### 3.5. Tongue-Lip Motor Function in Frailty

Oral diadochokinesis (ODK) was assessed as tongue-lip motor function in two studies and reported for men and women together [19,20]. The numbers of repetitions per second were significantly fewer in individuals with frailty than in those without frailty for /pa/ (MD −0.46 times/s, 95% CI −0.68 to −0.24, I^2^ = 0%; two studies), /ta/ (MD −0.62 times/s, 95% CI −0.91 to −0.33, I^2^ = 22%; two studies), and /ka/ (MD −0.54 times/s, 95% CI −0.92 to −0.17, I^2^ = 37%; two studies) according to the results of CM. However, the results of BM were inconclusive for /pa/ (MD −0.48, 95% CrI −1.10 to 0.11), /ta/ (MD −0.63, 95% CrI −1.29 to 0.00), and /ka/ (MD −0.54, 95% CrI −1.28 to 0.12) (Figure S5). Watanabe et al. assessed ODK according to sex, and showed that individuals with frailty had significantly fewer repetitions per second than robust men (/pa/; MD −0.60 times/s, 95% CI −0.74 to −0.46, /ta/; MD −0.60 times/s, 95% CI −0.74 to −0.46, and /ka/; MD −0.60 times/s, 95% CI −0.75 to −0.45) and women (/pa/; MD −0.70 times/s, 95% CI −0.83 to −0.57, /ta/; MD −0.70 times/s, 95% CI −0.84 to −0.56, and /ka/; MD −0.70 times/s, 95% CI −0.84 to −0.56) [28]. Three studies categorized ODK as higher and lower ODK, which was defined at a cut-off of <6 repetitions per second for /pa/, /ta/, or /ka/, and reported that for men and women together [19,22,23]. The OR for low ODK was higher in individuals with frailty than in those without frailty, according to the results of both CM (OR 1.73, 95% CI 1.19 to 2.51, I^2^ = 0%, three studies) and BM (OR 0.55, 95% CrI 0.01 to 1.08) (Figure S6). Yoshida et al. reported the numbers of repetitions in individuals with pre-frailty and showed no significant difference on comparison with robust individuals (/pa/; MD 0.00 times/s, 95% CI −0.14 to 0.14, /ta/; MD 0.00 times/s, 95% CI −0.16 to 0.16, and /ka/; MD 0.00 times/s, 95% CI −0.15 to 0.15) [20]. Watanabe et al. assessed ODK according to sex, and the number of repetitions was significantly lower in men with pre-frailty (/pa/; MD −0.20 times/s, 95% CI −0.28 to −0.12, /ta/; MD −0.10 times/s, 95% CI −0.18 to −0.02, and /ka/; MD −0.10 times/s, 95% CI −0.19 to −0.01) and women with pre-frailty (/pa/; MD −0.20 times/s, 95% CI −0.28 to −0.12, /ta/; MD −0.20 times/s, 95% CI −0.28 to −0.12, and /ka/; MD −0.20 times/s, 95% CI −0.27 to −0.13) than in robust individuals [28]. Shimazaki et al. assessed individuals with low ODK and found that individuals with pre-frailty had higher OR for low ODK, which was defined as <6 repetitions per second for /pa/, /ta/, or /ka/ (OR 1.54, 95%CI 1.06 to 2.24) [23].

### 3.6. Tongue-Lip Motor Function in Sarcopenia

Two studies reported ODK for men and women together [20,25]. Kugimiya et al. reported the number of repetitions as a median value with IQR [25], which we converted into a mean value with SD for meta-analyses. The number of repetitions per second in ODK for /pa/, /ta/, and /ka/ was significantly fewer in individuals with sarcopenia than in those without sarcopenia, according to the results of CM (/pa/; MD −0.37 times/s, 95% CI −0.50 to −0.25, I^2^ = 0%, /ta/; MD −0.38 times/s, 95% CI −0.50 to −0.27, I^2^ = 0%, and /ka/; MD −0.38 times/s, 95% CI −0.49 to −0.27, I^2^ = 0%; two studies). However, the results of BM were inconclusive for /pa/ (MD −0.37, 95% CrI −0.87 to 0.17), /ta/ (MD −0.37, 95% CrI −0.88 to 0.17), and /ka/ (MD −0.37, 95% CrI −0.87 to 0.17) (Figure S7). Suzuki et al. reported ODK in women only; those with sarcopenia had fewer repetitions per second in ODK than robust women, who were categorized into non-pre-sarcopenia, non-dynapenia, and non-sarcopenia (/pa/; MD −0.37 times/s, 95%CI −0.50 to −0.25, I^2^ = 0%, /ta/; MD −0.38 times/s, 95% CI −0.50 to −0.27, I^2^ = 0%, and /ka/; MD −0.38 times/s, 95% CI −0.49 to −0.27, I^2^ = 0%) [27]. No studies assessed ODK as a category of low ODK.

### 3.7. Presence of Dysphagia in Frailty

**Figure 4:**
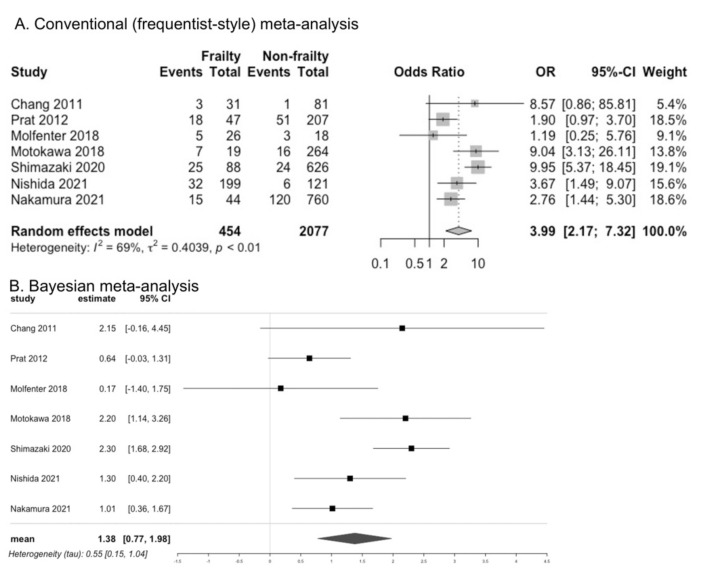
Comparison of presence of dysphagia between individuals with and without frailty [22,23,32,33,34,35,36].

Seven studies assessed the association between dysphagia and frailty [22,23,32,33,34,35,36]; the OR for dysphagia was higher in individuals with frailty than in those without frailty, according to the results of both CM (OR 3.99, 95% CI 2.17 to 7.32, I^2^ = 69%, seven studies) and BM (OR 1.38, 95% CrI 0.77 to 1,98) (Figure 4). One study (Weiss et al.) assessed swallowing function using the time to drink water, volume swallowed per second, and the number of swallows in a water swallow test and showed reduced swallowing function in individuals with frailty compared to those without frailty [37]. Three studies assessed the association between dysphagia and pre-frailty [23,32,35]; the OR was not significant in comparison with robust individuals, according to the results of CM (OR 2.24, 95% CI 0.56 to 8.98, I^2^ = 82%, three studies), and the results of BM were inconclusive (OR 1.00, 95% CrI −0.18 to 2.00) (Figure S8).

### 3.8. Presence of Dysphagia in Sarcopenia

**Figure 5:**
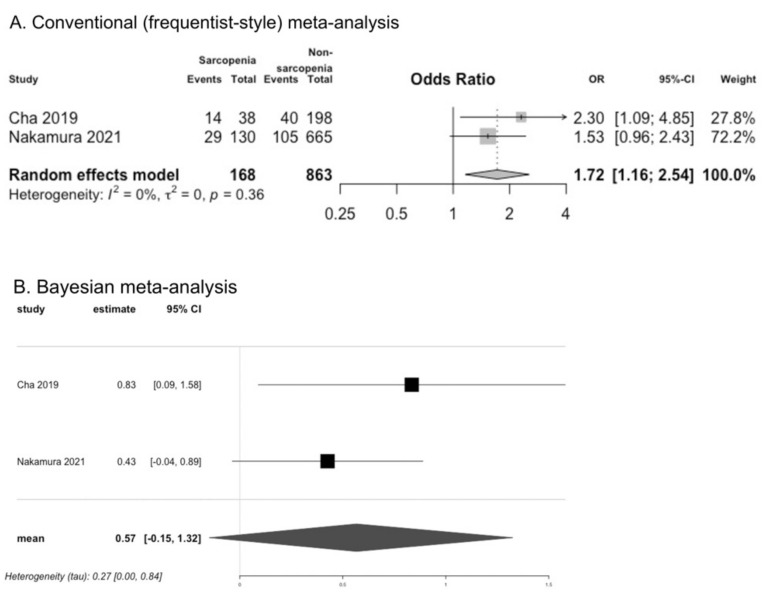
Comparison of presence of dysphagia between individuals with and without sarcopenia [22,38].

Two studies assessed the association between dysphagia and sarcopenia [22,38]; the OR for dysphagia was significantly higher in individuals with sarcopenia than in those without sarcopenia, according to the results of CM (OR 1.72, 95% CI 1.16 to 2.54, I^2^ = 0%; two studies). However, the results of BM were inconclusive (OR 0.57, 95% CrI −0.15 to 1.32) (Figure 5). Three studies reported EAT-10 scores [20,24,25]. We converted the median value with IQR of EAT-10 scores obtained from one study (Kugimiya et al.) into the mean value with SD [25]. In these studies, the difference in the EAT-10 scores between individuals with and without sarcopenia was not significant according to the results of CM (MD −0.28, 95% CI −0.77 to 0.22, I^2^ = 77%; three studies), and the results of BM were inconclusive (MD −0.30, 95% CrI −0.90 to 0.37) (Figure S9). Suzuki et al. analyzed the association between EAT-10 scores and sarcopenia in women and found no significant difference in EAT-10 scores between women with sarcopenia (median 1.0, IQR 0–3.5) and robust women (median 0, IQR 0–2.0) [27].

### 3.9. Development of Frailty, Sarcopenia, and Disability

Table 3 shows the prospective associations between oral function and dysphagia at baseline and frailty, sarcopenia, and disability at follow-up in four prospective cohort studies [7,39,40,41].

#### 3.9.1. Frailty

Occlusal force was not a statistically significant variable for worsening frailty status, which was defined as the change from robust to pre-frail, pre-frail to frail, or robust to frail status, during a 2-year follow-up in one study (Horibe et al.) [39]. Iwasaki et al. reported that individuals with lower occlusal force (men: ≤145 N, women: ≤108 N) significantly developed frailty during a 5-year follow-up [41]. These two studies did not assess tongue pressure, tongue-lip motor function, and dysphagia. Takeuchi et al. showed that the number of repetitions in ODK for /ta/ was a significant factor for developing frailty during a 2-year follow-up after adjusting for occlusal force, tongue pressure, EAT-10 score, ODK for /pa/ and /ka/, and other potential factors [40]. Tanaka et al. showed that low occlusal force, ODK for /ta/, and tongue pressure at baseline were significant factors for the development of frailty during a 2-year follow-up after adjusting for potential factors [7].

#### 3.9.2. Sarcopenia

Tanaka et al. investigated the association between decreased oral function and dysphagia at baseline and the presence of sarcopenia at follow-up and showed that low tongue pressure was a significant factor for the presence of sarcopenia after a 2-year follow-up [7].

#### 3.9.3. Disability

Tanaka et al. showed an association between oral function and dysphagia at baseline and the presence of physical dependence at follow-up, which was defined as the need for care in all activities of daily life [7]. They showed that ODK for /ta/ and dysphagia at baseline were significant factors for the presence of physical dependence after a 4-year follow-up.

### 3.10. Study Quality and Certainty of Evidence

In the study quality assessment using the National Institutes of Health’s Quality Assessment Tool for Observational Cohort and Cross-Sectional Studies, oral function, dysphagia, and physical function were assessed by blinded assessors in only one study (Nakamura et al.) [22]. No other study had reported blindness for the assessments. We evaluated question 14 of the assessment of prospective cohort studies as “cannot determine” because we did not find the reason for the selection of potential covariates in the models. The quality assessment results are presented in Table 4. We rated the certainty of evidence for the prospective associations as very low due to the insufficient information on the adjustment for potential covariates assessed in the risk of bias domain and the lack of information on the outcome events leading to imprecision in the evidence.

## 4. Discussion

This review investigated the association of oral function and dysphagia with frailty and sarcopenia in community-dwelling older adults. Both conventional (frequentist-style) meta-analysis (CM) and Bayesian meta-analyses (BM) showed lower tongue pressure in individuals with frailty and those with sarcopenia and higher OR for dysphagia and lower tongue-lip motor function in individuals with frailty. The certainty of evidence of prospective associations of oral function and dysphagia with adverse outcomes was low because of insufficient information and a limited number of studies.

Most of the included studies were cross-sectional, and only four were prospective cohort studies. Although most studies did not report the blindness of the assessors, overall study quality was good. We included the cross-sectional studies in the meta-analyses for tongue pressure, tongue-lip motor function, and dysphagia in individuals with frailty and sarcopenia. Meta-analysis for occlusal force was conducted only in studies that included individuals with sarcopenia, and meta-analysis for suprahyoid muscle strength was not conducted, neither in studies that included individuals with frailty nor in those with sarcopenia. Overall, the number of studies investigating oral function in individuals with pre-frailty was insufficient to conduct meta-analyses. However, we could show an association between pre-frailty and dysphagia using four studies in the meta-analysis. Future cross-sectional studies investigating oral function, especially in individuals with pre-frailty, should be performed to understand oral function in community-dwelling older adults. Regarding the prospective association of oral function and dysphagia at baseline with frailty and sarcopenia at follow-up, adequate studies did not exist to determine these associations. We did not find any study that investigated oral function and dysphagia at baseline and adverse outcomes such as malnutrition, aspiration pneumonia, suffocation, and death. Interestingly, no study has investigated the association between frailty or sarcopenia at baseline and oral function and dysphagia at follow-up. This might suggest that oral and swallowing problems are considered a cause of frailty and sarcopenia. Future studies investigating these associations may be useful in community healthcare practice. Most included studies were conducted in Japan. Therefore, more studies should be conducted worldwide to understand the physiology of older adults globally.

Some differences in the results between CM and BM were evident, including the outcomes of occlusal force and dysphagia in individuals with sarcopenia, tongue pressure in individuals with pre-frailty, and ODK for the syllables in individuals with frailty and sarcopenia. These differences may indicate the instability of the results, which may have been caused by inter-study heterogeneity and the limited number of included studies. However, these outcomes may be useful for focusing on or increasing the robustness of the results in future studies.

Considerable heterogeneity was evident in four meta-analyses. The first meta-analysis was performed to compare the tongue pressure between individuals with pre-frailty and robust individuals using two studies. Both studies used the same criteria for defining frailty; however, the study participants in the study by Yamanashi et al. were from a community cohort [21], while Yoshida et al. included participants who attended a physical fitness assessment at a university [20]. The differences in the characteristics of included participants might have caused the heterogeneity. The second meta-analysis used two studies to compare tongue pressure between women with and without sarcopenia. Machida et al. categorized participants as those with and without sarcopenia [26], and Suzuki et al. categorized participants as those with sarcopenia, dynapenia, pre-sarcopenia, or robustness [27]. We categorized robust participants in the “non-sarcopenia” group in the meta-analysis; this resulted in one study (Suzuki et al., 2018) showing a greater difference in tongue pressure between individuals with and without sarcopenia. The third meta-analysis was performed to compare the presence of dysphagia between individuals with pre-frailty and robust individuals using three studies. In one study (Chang et al.), the 95% CI of OR for dysphagia was wide [32]. This may have been caused by the use of an assessment tool for dysphagia, which was mentioned just as “self-reported”. In another study (Shimazaki et al.), the OR for dysphagia was high in individuals with pre-frailty [23]. However, we could not postulate the associated reasons. The last meta-analysis was performed to compare the EAT-10 scores between people with and without sarcopenia using three studies. A higher EAT-10 score indicated more symptoms of dysphagia. However, Yoshida et al. showed lower EAT-10 scores in individuals with sarcopenia than in those without sarcopenia [20], but we could not postulate the reason for this result. This could have led to a greater difference in EAT-10 score than that in the other two studies.

Only a few reviews have assessed the association of dysphagia with either frailty or sarcopenia [42,43]. These reviews did neither perform a meta-analysis for oral function and BM for dysphagia nor focus on community-dwelling older adults. Moreover, these reviews focused on either sarcopenia or frailty. Our review included both sarcopenia and frailty to understand the bigger picture of oral function and dysphagia in community-dwelling older adults. As a limitation, we included articles in English or Japanese only. Therefore, a selection bias is possible. However, this may not have significantly affected our results because only a few studies have been conducted outside Japan. In addition, we used the Japanese database, which might have rather increased the number of studies included in this review. Second, we did not use databases other than those described in the Methods section. This may also have led to a selection bias, although we searched for relevant literature manually as well as by interrogating researchers. Third, we conducted the meta-analyses regardless of the definitions of frailty and sarcopenia, which might cause some heterogeneity in the meta-analyses. However, this might not affect the results significantly because all assessment tools used for frailty and sarcopenia in the included studies have been validated. Fourth, most studies included in the meta-analyses were conducted in Japan. Thus, our results should be interpreted carefully, especially for older adults outside of Asia. Lastly, the clinical importance of the differences in oral function and dysphagia detected by the meta-analysis is unclear. Whether the differences detected in our review are important in community-dwelling older adults should be determined in future research. References [44,45,46,47,48,49,50,51,52,53,54,55,56,57,58,59,60,61,62,63,64,65,66,67,68,69,70] are cited in Supplementary Materials.

## 5. Conclusions

Community-dwelling older adults with frailty had lower oral function and dysphagia, and those with sarcopenia had lower oral function. Decreased oral function and the presence of dysphagia can be important characteristics of frailty and sarcopenia. Therefore, we should focus on the assessment of these characteristics along with physical function in community-dwelling older individuals. More longitudinal studies investigating the association of oral function and dysphagia with adverse outcomes and studies conducted in various countries are needed in the future.

## Figures and Tables

**Table 1:** Study characteristics.

	Country	Study Year	Study Design	Mean Age (SD) of All Participants	Number of Participants (Men, %)	Prevalence of Frailty and Sarcopenia, *n* (%)
Cha et al., 2019	Seongnam, Korea	2005–2006	Cross-sectional	76.6 (5.8)	236 (114, 48.3)	Pre-frailty: NR Frailty: NR Sarcopenia: 38 (16.1)
Chang et al., 2011	Taipei, Taiwan	NR	Cross-sectional	71.1 (3.8)	275 (127, 46.2)	Pre-frailty: 161 (58.5) Frailty: 31 (11.3) Sarcopenia: NR
Chen et al., 2020	Taipei, Taiwan	NR	Cross-sectional	75.1 (5.8)	94 (26, 27.7)	Pre-frailty: NR Frailty: NR Sarcopenia: 47 (50)
Horibe et al., 2018	Tokyo, Japan	2014	Cross-sectional	72.7 (5.2)	659 (264, 40.1)	Pre-frailty: 220 (33.4) Frailty: 92 (14.0) Sarcopenia: NR
Horibe et al., 2018	Tokyo, Japan	2013–2015	Prospective cohort	NR	418 (175, 41.9)	Pre-frailty: NR Frailty: baseline 62 (7.9%), follow-up 113 (14.1) Sarcopenia: NR
Iwasaki et al., 2018	Niigata, Japan	2003–2008	Prospective cohort	75 (0)	322 (181, 56.2)	Pre-frailty: NR Frailty: baseline 0, follow-up 49 (15.2) Sarcopenia: NR
Kera et al., 2017	Tokyo, Japan	2011–2013	Cross-sectional	NR	1380 (592, 42.9)	Pre-frailty: NR Frailty: 369 (26.7) Sarcopenia: NR
Kugimiya et al., 2021	Tokyo, Japan	2018	Cross-sectional	76.7 (8.4)	871 (268, 30.8)	Pre-frailty: NR Frailty: NR Sarcopenia: 163 (18.7)
Machida et al., 2017	NR, Japan	NR	Cross-sectional	NR	197 (97, 49.2)	Pre-frailty: NR Frailty: NR Sarcopenia: 68 (34.5)
Molfenter et al., 2018	New York, U.S.	NR	Cross-sectional	76.9 (7.1)	44 (21, 47.7)	Pre-frailty or frailty: 26 (59.1) Sarcopenia: NR
Motokawa 2018	Saitama, Japan	2014	Cross-sectional	69.6 (NR)	283 (121, 42.8)	Pre-frailty: 165 (58.3) Frailty: 19 (6.7) Sarcopenia: NR
Murakami et al., 2014	Tokyo, Japan	2012	Cross-sectional	73.0 (5.1)	761 (314, 41.3)	Pre-frailty: NR Frailty: NR Sarcopenia: 116 (15.2)
Nishida et al., 2021	Niigata, Japan	2018–2019	Cross-sectional	77.3 (6.6)	320 (52, 16.3)	Pre-frailty: 154 (48.1) Frailty: 45 (14.1) Sarcopenia: NR
Nakamura et al., 2021	Kagoshima, Japan	2018	Cross-sectional	74.9 (6.29)	832 (303, 36.4)	Pre-frailty: NR Frailty: 44 (5.5) Sarcopenia: 130 (16.4)
Serra-Prat et al., 2012	Barcelona, Spain	NR	Cross-sectional	78.2 (5.6)	254 (136, 53.5)	Pre-frailty: NR Frailty: 46 (18.1) Sarcopenia: NR
Suzuki et al., 2018	NR, Japan	NR	Cross-sectional	(Median [IQR]: 81.0 [75.0–85.0])	245 (0, 0)	Pre-frailty: NR Frailty: NR Sarcopenia: 29 (11.8)
Satake et al., 2019	Aomori, Japan	2016	Cross-sectional	74.4 (7.8)	467 (173, 37.0)	Pre-frailty: NR Frailty: 47 (10.1) Sarcopenia: NR
Shimazaki et al., 2020	Aichi, Japan	2018	Cross-sectional	NR	978 (463, 47.3)	Pre-frailty: 295 (30.2) Frailty: 81 (8.3) Sarcopenia: NR
Takeuchi et al., 2022	Okayama, Japan	2017–2021	Prospective cohort	71.9 (5.4)	97 (34, 35.1)	Pre-frailty: NR Frailty: baseline 0 (0), follow-up 34 (35.1) Sarcopenia: NR
Tanaka et al., 2018	Chiba, Japan	2012–2016	Prospective cohort	73.0 (5.5)	2011 (NR, 50)	Pre-frailty: NR Frailty: baseline 0 (0), follow-up 83 (7.2) Sarcopenia: baseline 0 (0%), follow-up 63 (5.2)
Watanabe et al., 2017	Aichi, Japan	2011–2012	Cross-sectional	72.1 (5.6)	4720 (2274, 48.2)	Pre-frailty: 2691 (57.0) Frailty: 535 (11.3) Sarcopenia: NR
Weiss 2021	Israel	NR	Cross-sectional	NR	180 (74, 41.1)	Pre-frailty: 67 (37.2) Frailty: 20 (11.2) Sarcopenia: NR
Yoshida et al., 2022	Kyoto, Japan	2019	Cross-sectional	75.0 (NR)	340 (69, 20.3)	Pre-frailty: 155 (46.8) Frailty: 9 (2.7) Sarcopenia: 35 (10.3)
Yamanashi et al., 2018	Nagasaki, Japan	2014–2015	Cross-sectional	72.8 (7)	1603 (650, 40.5)	Pre-frailty: 605 (37.7) Frailty: 30 (1.9) Sarcopenia: NR

NR, not reported; SD, standard deviation; IQR, interquartile range.

**Table 2:** Items assessed and tools used in studies.

	**Frailty**	**Sarcopenia**	**Tongue Pressure**	**Occlusal Force**	**Suprahyoid Muscle Strength**	**Tongue-Lip Motor Function “pa”**	**Tongue-Lip Motor Function “ta”**	**Tongue-Lip Motor Function “ka”**	**Overall Tongue-Lip Motor Function**	**Dysphagia**
Cha et al., 2019		◯ AWGS 2014								◯ SSA
Chang et al., 2011	◯ CHS									◯ Self-reported
Chen et al., 2020		◯ AWGS 2014	◯							◯ VF, EAT-10, WST
Horibe et al., 2018 a	◯ KCL			◯						
Horibe et al., 2018 b	◯ KCL			◯						
Iwasaki et al., 2018	◯ CHS			◯						
Kera 2016	◯ KCL			◯						
Kugimiya et al., 2021		◯ AWGS 2019	◯	◯		◯	◯	◯		◯ EAT-10
Machida et al., 2017		◯ AWGS 2014	◯		◯					
Molfenter et al., 2018	◯ CHS									◯ VF
Motokawa 2018	◯ J-CHS									◯ Seirei-Questionnaire
Murakami et al., 2014		◯ AWGS 2014		◯						
Nakamura et al., 2021	◯ CHS	◯ AWGS 2014	◯						◯	◯ EAT-10
Nishida et al., 2021	◯ CHS									◯ EAT-10
Serra-Prat 2012	◯ CHS									◯ V-VST
Suzuki et al., 2018		◯ AWGS 2014	◯			◯	◯	◯		◯ EAT-10
Satake et al., 2019	◯ FRAIL scale		◯			◯	◯	◯	◯	
Shimazaki et al., 2020	◯ KCL		◯	◯					◯	◯ EAT-10
Takeuchi et al., 2022	◯ J-CHS		◯	◯		◯	◯	◯		◯ EAT-10
Tanaka et al., 2018	◯ CHS	◯ AWGS 2014	◯	◯		◯	◯	◯		◯ Self-reported
Watanabe et al., 2017	◯ CHS			◯		◯	◯	◯		
Weiss 2021	◯ FRAIL scale									◯ TWST
Yoshida et al., 2022	◯ CHS and KCL	◯ AWGS 2019	◯	◯		◯	◯	◯		◯ EAT-10
Yamanashi et al., 2018	◯ CHS		◯							

AWGS, Working Group for Sarcopenia; CHS, Cardiovascular Health Study; J-CHS, Japanese version of the CHS; EAT-10, 10-item Eating Assessment Tool; KCL, Kihon Checklist; SSA, Standardized Swallowing Assessment; TWST, Timed Water Swallow Test; VF, videofluoroscopy; WST, Water Swallowing Test; V-VST, Volume-Viscosity Swallow Test. Note: The blank columns indicate “not-assessed”. Overall tongue-lip motor function refers to the combined assessments of /pa/, /ta/, and /ka/.

**Table 3:** Association between oral function and dysphagia at baseline and frailty, sarcopenia, and disability at follow-up.

	Follow-Up Period	Assessed Items at Baseline	Frailty	Sarcopenia	Disability
Horibe et al., 2018	2 years	Occlusal force	Adjusted OR (95% CI) of low occlusal force for frailty progression (from healthy to pre-frail, from pre-frail to frail, or from healthy to frail): 1.00 (0.99 to 1.00)	NR	NR
Iwasaki et al., 2018	5 years	Occlusal force	Adjusted HRs (95% CI) of low occlusal force: 2.78 (1.15 to 6.72)	NR	NR
Takeuchi et al., 2022	2 years	Tongue-lip motor function: ODK for /ta/	Adjusted OR (95% CI) of ODK for /ta/: 1.85 (1.02 to 3.35). ODK for /ka/ and /pa/: Not significant.	NR	NR
Tanaka et al., 2018	Frailty and sarcopenia: 2 years Disability: 45 months	Occlusal force Tongue-lip motor function; /pa/, /ta/, and /ka/ Tongue pressure Dysphagia	Low maximum occlusal force: 29% (*p* = 0.017) Low /pa/: 26% (*p* = 0.321) Low /ta/: 29% (*p* = 0.021) Low /ka/: 20% (*p* = 0.598) Low tongue pressure: 26% (*p* = 0.037) Presence of dysphagia: 25% (*p* = 0.094)	Low maximum occlusal force: 26% (*p* = 0.221) Low /pa/: 21% (*p* = 0.791) Low /ta/: 27% (*p* = 0.083) Low /ka/: 14% (*p* = 0.554) Low tongue pressure: 30% (*p* = 0.039) Presence of dysphagia: 26% (*p* = 0.078)	Low maximum occlusal force: 32% (*p* = 0.066) Low /pa/: 23% (*p* = 0.354) Low /ta/: 22% (*p* = 0.011) Low /ka/: 19% (*p* = 0.637) Low tongue pressure: 28% (*p* = 0.051) Presence of dysphagia: 23% (*p* = 0.036)

NR, not reported; OR, odds ratio; CI, confidence interval; HR, hazards ratio; ODK, oral diadochokinesis. Note: Types of models and covariates in the models used in the studies. Iwasaki et al., 2018: Covariates; sex, depression, diabetes; Eichner index model: Cox proportional-hazards regression analysis. Horibe et al., 2018b: Covariates; age, sex, number of teeth, hand grip, walking speed, Mini-Mental State Examination score, self-reported depression scale, skeletal muscle mass index, number of medications taken; Model: binomial logistic regression analysis (forced entry method). Takeuchi et al., 2022: Covariates; number of teeth, clinical attachment level, /ta/ sound, /ka/ sound; Model: binomial logistic regression analysis (variable reduction method). Tanaka et al., 2018: Covariates: age, sex, body mass index, chronic conditions, depressive symptoms, cognitive function, living arrangement, yearly income, and smoking behavior; Model: Cox proportional-hazards regression analysis. Low indicates less than the first quantile of the data. Percentage indicates the proportion of individuals with low function who developed an outcome.

**Table 4:** Quality assessment for each study using the National Institutes of Health’s Quality Assessment Tool for Observational Cohort and Cross-Sectional Studies.

	1	2	3	4	5	6	7	8	9	10	11	12	13	14	D
Cha et al., 2019	*	*	*	*	?	NA	NA	NA	*	NA	*	?	NA	NA	✢
Chang et al., 2011	*	*	*	*	?	NA	NA	*	*	NA	CD	?	NA	NA	✢
Chen et al., 2020	*	*	*	*	*	NA	NA	NA	*	NA	*	?	NA	NA	✢
Horibe et al., 2018 a	*	*	*	*	?	NA	NA	*	*	NA	*	?	NA	NA	✢
Horibe et al., 2018 b	*	*	*	*	?	*	*	NA	*	−	*	?	−	CD	→
Iwasaki et al., 2017	*	*	*	*	?	*	*	*	*	−	*	?	*	CD	→
Kera 2016	*	*	*	*	?	NA	NA	*	*	NA	*	?	NA	NA	✢
Kugimiya et al., 2021	*	*	*	*	?	NA	NA	NA	*	NA	*	?	NA	NA	✢
Machida et al., 2017	*	*	*	*	?	NA	NA	NA	*	NA	NA	?	NA	NA	✢
Molfenter et al., 2018	*	*	*	*	?	NA	NA	*	*	NA	*	?	NA	NA	✢
Motokawa 2018	*	*	*	*	?	NA	NA	*	*	NA	*	?	NA	NA	✢
Murakami et al., 2014	*	*	*	*	?	NA	NA	NA	*	NA	*	?	NA	NA	✢
Nishida et al., 2021	*	*	*	*	?	NA	NA	*	*	NA	*	?	NA	NA	✢
Nakamura et al., 2021	*	*	?	*	?	NA	NA	−	*	NA	*	*	NA	NA	✢
Serra-Prat 2012	*	*	*	*	*	NA	NA	−	−	NA	*	?	NA	NA	✢
Suzuki et al., 2018	*	*	?	*	?	NA	NA	NA	*	NA	*	?	NA	NA	✢
Satake et al., 2019	*	*	?	*	?	NA	NA	−	*	NA	*	?	NA	NA	✢
Shimazaki et al., 2020	*	*	*	*	?	NA	NA	*	*	NA	*	?	NA	NA	✢
Takeuchi et al., 2022	*	*	*	*	−	*	*	NA	*	*	*	?	*	CD	→
Tanaka et al., 2018	*	*	*	*	?	*	*	NA	*	*	*	?	*	CD	→
Watanabe et al., 2017	*	*	*	*	?	NA	NA	*	*	NA	*	?	NA	NA	✢
Weiss 2021	*	−	?	?	?	NA	NA	*	*	NA	*	?	NA	NA	✢
Yamanashi et al., 2018	*	*	*	*	?	NA	NA	*	*	NA	*	?	NA	NA	✢
Yoshida et al., 2022	*	*	?	*	*	NA	NA	*	*	NA	*	?	NA	NA	✢

*, yes; −, no; ?, not reported; ✢, cross-sectional study; →, prospective cohort study; NA, not applicable; CD, cannot determine; D, study design. Criterion 1: Was the research question or objective in this paper clearly stated? Criterion 2: Was the study population clearly specified and defined? Criterion 3: Was the participation rate of eligible persons at least 50%? Criteria 4: Were all subjects selected or recruited from the same or similar populations (including the same time period)? Were inclusion and exclusion criteria for participation in the study prespecified and applied uniformly to all participants? Criterion 5: Was sample size justification, power description, or variance and effect estimates provided? Criterion 6: For the analyses in this study, were the exposure(s) of interest measured before the outcome(s) being measured? Criterion 7: Was the timeframe sufficient for association between exposure and outcome to become evident, if it existed? Criterion 8: For exposures that can vary in amount or level, did the study examine different levels of exposure as related to the outcome (e.g., categories of exposure or exposure measured as continuous variables)? Criterion 9: Were the exposure measures (independent variables) clearly defined, valid, reliable, and implemented consistently across all study participants? Criterion 10: Was the exposure(s) assessed more than once over time? Criterion 11: Were the outcome measures (dependent variables) clearly defined, valid, reliable, and implemented consistently across all study participants? Criterion 12: Were the outcome assessors blinded to the exposure status of participants? Criterion 13: Was the patient lost to follow-up after a baseline of 20% or less? Criterion 14: Were key potential confounding variables measured and adjusted statistically for their impact on the relationship between exposure(s) and outcome(s)?

## Data Availability

All data are included in the article.

## Supplementary Materials

The following supporting information can be downloaded at: https://www.mdpi.com/article/10.3390/cells11142199/s1. Table S1: MOOSE checklist for the present review. Table S2. Search strategies. Table S3. Excluded studies and reasons. Table S4. Inclusion and exclusion criteria of included studies. Figure S1. PRISMA 2020 flow diagram. Figure S2. Comparison of low tongue pressure between individuals with and without frailty. Figure S3. Comparison of tongue pressure between individuals with pre-frailty and robust individuals. Figure S4. Comparison of tongue pressure between women with and without sarcopenia. Figure S5. Comparison of the number of repetitions in oral diadochokinesis between individuals with and without frailty. Figure S6. Comparison of low oral diadochokinesis in individuals with and without frailty. Figure S7. Comparison of the number of repetitions in oral diadochokinesis between individuals with and without sarcopenia. Figure S8. Comparison of dysphagia between individuals with pre-frailty and robust individuals. Figure S9. Comparison of Eating Assessment Tool-10 scores between individuals with and without sarcopenia. References [44,45,46,47,48,49,50,51,52,53,54,55,56,57,58,59,60,61,62,63,64,65,66,67,68,69,70] are cited in Supplementary Materials.
