# Supplementary material for: Association of Oral Function and Dysphagia with Frailty and Sarcopenia in Community-Dwelling Older Adults: A Systematic Review and Meta-Analysis
Source: Cells. 2022 Jul 14;11(14):2199. doi: 10.3390/cells11142199 (PMC9316124; doi:10.3390/cells11142199)
Supplement: Supplementary file 1 [file cells-11-02199-s001.zip › cells-1815068-supplementary.pdf]

Table S1. MOOSE checklist for the present review

Item No	Recommendation	Reported on Page No
Reporting of background should include		
1	Problem definition	2
2	Hypothesis statement	2
3	Description of study outcome(s)	2
4	Type of exposure or intervention used	2
5	Type of study designs used	2
6	Study population	2
Reporting of search strategy should include		
7	Qualifications of searchers (e.g., librarians and investigators)	2
8	Search strategy, including time period included in the synthesis and key words	2, Table S2
9	Effort to include all available studies, including contact with authors	2
10	Databases and registries searched	2
11	Search software used, name and version, including special features used (e.g., explosion)	2
12	Use of hand searching (e.g., reference lists of obtained articles)	2
13	List of citations located and those excluded, including justification	Tables S3 and S4
14	Method of addressing articles published in languages other than English	2
15	Method of handling abstracts and unpublished studies	2
16	Description of any contact with authors	2
Reporting of methods should include		
17	Description of relevance or appropriateness of studies assembled for assessing the hypothesis to be tested	2–3
18	Rationale for the selection and coding of data (e.g., sound clinical principles or convenience)	2–3
19	Documentation of how data were classified and coded (e.g., multiple raters, blinding, and interrater reliability)	3
20	Assessment of confounding (e.g., comparability of cases and controls in studies where appropriate)	NA
21	Assessment of study quality, including blinding of quality assessors, stratification, or regression on possible predictors of study results	3
22	Assessment of heterogeneity	4
23	Description of statistical methods (e.g., complete description of fixed or random effects models, justification of whether the chosen models account for	3–4

	predictors of study results, dose-response models, or cumulative meta-analysis) in sufficient detail to be replicated	
24	Provision of appropriate tables and graphics	NA
Reporting of results should include		
25	Graphics summarizing individual study estimates and overall estimate	Figure 1–5<br>Figure S2–8
26	Table summarizing descriptive information for each study included	Tables 1, 2, and S4
27	Results of sensitivity testing (e.g., subgroup analysis)	NA
28	Indication of statistical uncertainty of findings	8–12

**Item No**	**Recommendation**	**Reported on Page No**
Reporting of discussion should include		
29	Quantitative assessment of bias (e.g., publication bias)	NA
30	Justification for exclusion (e.g., exclusion of non-English language citations)	17
31	Assessment of quality of included studies	16
Reporting of conclusions should include		
32	Consideration of alternative explanations for observed results	18
33	Generalization of the conclusions (i.e., appropriate for the data presented and within the domain of the literature review)	18
34	Guidelines for future research	18
35	Disclosure of funding source	18

From: Stroup DF, Berlin JA, Morton SC, et al.: Meta-analysis of observational studies in epidemiology (MOOSE) group. Meta-analysis of observational epidemiological studies Proposal for reporting. *JAMA*. 2000;283(15):2008-2012. doi: 10.1001/jama.283.15.2008.

Table S2. Search strategies

PubMed	((((("sarcopenia"[Text Word] OR "muscular atrophy"[Text Word]) OR "muscle weakness"[Text Word]) OR "muscular weakness"[Text Word]) OR "frail*"[Text Word]) AND (((("community*"[Text Word] OR "healthy"[Text Word]) OR "healthy independent*"[Text Word]) OR "independent living"[MeSH Terms]) OR "healthy volunteers"[MeSH Terms])) AND ((((((((((("swallow*"[Text Word] OR "dysphagia"[Text Word]) OR "deglutition"[Text Word]) OR "deglutition disorders"[MeSH Terms]) OR "oral function"[Text Word]) OR "tongue"[Text Word]) OR "lip"[Text Word]) OR "suprahyoid"[Text Word]) OR "neck muscles"[MeSH Terms]) OR "pharyn*"[Text Word]) OR "videofluoroscopy"[Text Word]) OR "fiberoptic endoscopic evaluation of swallowing"[Text Word]) OR "oral muscle*"[Text Word])
Embase via ProQuest Dialog	S1: (EMB.EXACT.EXPLODE("sarcopenia") OR EMB.EXACT.EXPLODE("frailty") OR EMB.EXACT.EXPLODE("frail elderly") OR EMB.EXACT.EXPLODE("muscle atrophy") OR EMB.EXACT.EXPLODE("muscle weakness"))<br>S2: (TI,AB(sarcopenia OR frailty OR "frail elderly" OR "atrophy type 2" OR "atrophy, muscle" OR "degeneration, muscle" OR "muscle atrophy" OR "muscle cell degeneration" OR "muscle degeneration" OR "muscle fiber atrophy" OR "muscle fiber degeneration" OR "muscle recession" OR "muscle wasting" OR "muscular atrophy" OR "muscular degeneration" OR "muscular disorders, atrophic" OR "myofibrillar degeneration" OR "muscle atrophia" OR amyotrophia OR amyotrophy OR myoatrophy OR myodegeneration OR myophagism OR "muscle strength loss" OR "muscle weakening" OR "muscle weakness" OR "muscular insufficiency" OR "muscular weakness" OR "neuromuscular fatigue" OR "weakness, muscle"))<br>S3: (S1 or S2)<br>S4: (EMB.EXACT.EXPLODE("normal human") OR EMB.EXACT.EXPLODE("independent living") OR EMB.EXACT.EXPLODE("community dwelling person"))<br>S5: (TI,AB("healthy adult" OR "healthy human" OR "healthy humans" OR "healthy patient" OR "healthy people" OR "healthy person" OR "healthy subject" OR "healthy subjects" OR "healthy volunteer" OR "healthy volunteers" OR "human, normal" OR "normal human" OR "normal humans" OR "normal subject" OR "normal subjects" OR "normal volunteer" OR "normal volunteers" OR "independent living" OR "aging in place" OR "community dwelling individuals" OR "community dwelling participants" OR "community dwelling people" OR "community dwelling person" OR "community dwelling persons" OR "community dwelling subjects" OR "community-dwelling cohort" OR "community dwelling"))<br>S6: (S4 or S5)

	S7: (EMB.EXACT.EXPLODE("swallowing") OR EMB.EXACT.EXPLODE("dysphagia") OR EMB.EXACT.EXPLODE("tongue") OR EMB.EXACT.EXPLODE("suprahyoid muscle") OR EMB.EXACT.EXPLODE("neck muscle") OR EMB.EXACT.EXPLODE("pharynx") OR EMB.EXACT.EXPLODE("videofluoroscopic swallowing study") OR EMB.EXACT.EXPLODE("videofluoroscopic swallow study") OR EMB.EXACT.EXPLODE("fiberoptic endoscopic evaluation of swallowing"))<br>S8: (TI,AB(deglutition OR "oropharyngeal swallow" OR "reflex, deglutition" OR "swallow (deglutition)" OR "swallow (ingestion)" OR "swallow function" OR "swallow reflex" OR swallowing OR "swallowing reflex" OR aphagopraxia OR "deglutition disorder" OR "deglutition disorders" OR dysphagia OR dysphagias OR "swallowing disorder" OR "oral function" OR "oral muscle" OR lip OR lingua OR lingual OR tongue OR "tongue pressure" OR "cervical muscle" OR "muscle, neck" OR "muscle, nuchal" OR "neck muscle" OR "neck muscles" OR "nuchal muscle" OR "pharyngeal cavity" OR "pharyngeal wall" OR pharynx))<br>S9: (S7 or S8)<br>S10: (S3 and S6 and S9)<br>S11: (ANIMAL(Yes) NOT HUMAN(Yes))<br>S12: (S10 not S11)<br>S13: (rtype.exact("Conference Review" OR "Conference Abstract" OR "Conference Paper"))<br>S14: (S12 not S13)<br>S15: (S14 and (la.exact("English" OR "Japanese")) and (FAV(<=20220201)))
Ichu-Shi web [Japanese]	(((((筋肉減少症/TH or サルコペニア/AL)) or (((虚弱高齢者/TH or フレイル/AL) or (フレイル/TH or フレイル/AL)))) and ((地域/AL) or (健常/AL) or (健常高齢者/AL)) and (((嚥下/TH or 嚥下/AL)) or ((口/TH or 口腔/AL)) or ((舌/TH or 舌/AL)) or ((口唇/TH or 口唇/AL)) or ((舌骨/TH or 舌骨/AL)) or ((咽頭/TH or 咽頭/AL)) or (オーラル/AL) or ((咬合力/TH or 咬合力/AL))))) and ((PT=症例報告・事例除く) and (PT=原著論文) and CK=ヒト)
ClinicalTrials.gov	Condition or disease: sarcopenia OR frailty<br>Other terms: swallow* OR dysphagia OR oral function OR lip OR suprahyoid OR pharynx*<br>Study type: Observational study<br>Study Results: All studies
WHO-ICTRP	Condition (sarcopenia OR frailty) AND (swallow* OR dysphagia OR oral function OR tongue OR lip OR suprahyoid OR pharyn*)

WHO-ICTRP: The world health organization international clinical trials platform search portal

Table S3. Excluded studies and reasons

Albani et al. 2021 [44]	No data on oral and swallowing function based on frailty and sarcopenia status
Baba et al. 2022 [45]	No data on oral and swallowing function based on frailty and sarcopenia status
Bahat et al. 2019 [46]	Ineligible patient population
Buehring et al. 2013 [47]	No data on oral and swallowing function based on frailty and sarcopenia status
Chang et al. 2021[48]	No data on oral and swallowing function based on frailty and sarcopenia status
Chaleekrua et al. 2021 [49]	No data on the assessment of frailty and sarcopenia
Fernández et al. 2014 [50]	Ineligible patient population; least disabled women
Hironaka et al. 2020 [51]	No data on oral and swallowing function based on frailty and sarcopenia status
Hoshino et al. 2021 [52]	No data on the assessment of frailty and sarcopenia
Iwasaki et al. 2020a [53]	No data on the assessment of frailty and sarcopenia
Iwasaki et al. 2020b [54]	No data on the assessment of frailty and sarcopenia
Iwasaki et al. 2021 [55]	No data on the assessment of frailty and sarcopenia
Kaji et al. 2018 [56]	Ineligible patient population
Kugimiya et al. 2021[57]	No data on the assessment of frailty and sarcopenia
Koyama et al. 2022 [58]	No detail information on oral and swallowing function
Molfenter et al. 2019 [59]	No data on oral and swallowing function based on frailty and sarcopenia status
Morishita et al. 2021 [60]	No data on the assessment of frailty and sarcopenia
Murotani et al. 2021 [61]	No data on the assessment of frailty and sarcopenia
Nakao et al. 2021 [62]	No data on the assessment of frailty and sarcopenia
Nishida et al. 2020 [63]	No data on oral and swallowing function based on frailty and sarcopenia status
Nishimoto et al. 2020 [64]	No data on the assessment of frailty and sarcopenia
Saito et al. 2021 [65]	No data on oral and swallowing function based on frailty and sarcopenia status
Suzuki et al. 2020 [66]	No data on the assessment of frailty and sarcopenia

Takeuchi et al. 2021 [67]	No data on the assessment of frailty and sarcopenia
Takehara et al. 2021 [68]	No data on oral and swallowing function based on frailty and sarcopenia status
Tanaka et al. 2021 [69]	No data on the assessment of frailty and sarcopenia
Yamada et al. 2017 [70]	No data on oral and swallowing function based on frailty and sarcopenia status

Table S4. Inclusion and exclusion criteria of included studies

	Inclusion criteria	Exclusion criteria
Cha et al. 2019	Older adults living in Seongnam City	People with a history of diseases such as stroke, Parkinson’s disease, chronic obstructive pulmonary disease, temporomandibular joint disorder, and cognitive impairment defined as Mini-Mental State Examination (MMSE) score <23.
Chang et al. 2011	Community-dwelling residents aged 65–79 years in the 31 administrative areas of Toufen, who were selected randomly according to the population size of each administrative area.	Category 1, 2, or 7 in the Canadian study of health and aging clinical frailty scale<br>telephone version in Chinese.<br>1 or 2: very healthy<br>7: very sick
Chen et al. 2020	Community-dwelling older adults aged ≥65 years living in Wanhua District, Taipei City, Taiwan.	People who scored ≥3 on the EAT-10, those with a history of a neurological disorder such as cerebrovascular diseases, Parkinson’s disease, motor neuron disease, multiple sclerosis, myopathy, and head and neck cancers.
Horibe et al. 2018a	People aged 65–84 years living in nine districts of Itabashi-ku, Tokyo, Japan	Nursing home residents, those who did not answer all study questions, those with MMSE score <22, those receiving support from the Japanese long-term care insurance system, or those taking medicine for Parkinson’s disease, depression, and dementia.

Horibe et al. 2018b	People aged 65–86 years, living in one of nine districts of Itabashi-ku, Tokyo, Japan.	People living in nursing homes, with frailty, who received certification for the need for long-term care, with MMSE score <24, who were taking drugs for dementia, depression or Parkinson disease, or those in whom grip strength or walking speed could not be measured.
Iwasaki et al. 2018	Randomly sampled community-dwelling people aged 70 years living in Niigata City, Japan.	People with frailty at baseline and non-participation in any of the subsequent annual examinations
Kera et al. 2017	Older adults living in nine regions in Itabashi-ku, Tokyo	People living in nursing homes
Kugimiya et al. 2021	People aged ≥65 years living in Itabashi city, Tokyo.	People with missing survey data
Machida et al. 2017	Community-dwelling older adults, living independently and attending rehabilitation service facilities for care prevention	People having difficulty with communication or physical function, with dysphagia or the 10-item Eating Assessment Tool (EAT-10) score of 3 points or higher, malnutrition or at-risk status on the Mini-Nutritional Assessment short form, or history of stroke or neurodegenerative disease.
Molfenter et al. 2018	Older adults aged ≥65 years and in general good health who were recruited from local senior centers.	People with history of dysphagia, neurological disease, or head and neck cancer and surgery (other than routine dental/tonsil/adenoid surgeries).
Motokawa et al. 2018	Older adults aged 65–74 years who lived independently.	People who used a full or partial denture for front teeth.
Murakami et al. 2014	Older adults aged 65–85 years who lived in nine regions in Itabashi city, Tokyo, Japan.	People living in nursing homes, or people with heart pacemakers and/or walking difficulties.
Nakamura et al. 2021	People aged ≥65 years enrolled in Tarumizu Study	People who were hospitalized or lived at a care facility
Nishida et al. 2021	Consecutively recruited community-dwelling older adults aged ≥65 years who were participating in voluntary circle activities for preventive care and health promotion at public halls.	Not described.

Prat et al. 2012	People aged ≥70 years who were randomly selected from the Cirera-Molins Primary Care Centre database (Mataró, Barcelona, Spain).	Institutionalized people or those in palliative care or with a life expectancy < 3 months.
Suzuki et al. 2018	Women aged ≥65 years with ability to walk independently.	People with severe cardiac, pulmonary, or musculoskeletal disorders, stroke, Parkinson’s disease, head and neck cancers, neuromuscular diseases, severe cognitive and psychiatric impairment, or dysphagia.
Satake et al. 2019	Community-dwelling people aged ≥60 years	People with a medical history of cancer, stroke, ischemic heart disease, or Parkinson’s disease.
Shimazaki et al. 2020	Independent people aged 65–85 years living in Higashiura, Aichi Prefecture, Japan.	People who were unable to answer the questionnaire items due to cognitive decline.
Takeuchi et al. 2022	People aged ≥60 years who were able to understand the questionnaire, able to walk independently, able to visit the preventive clinic regularly, and judged robust on frailty assessment.	People without follow-up visits.
Tanaka et al. 2018	Community-dwelling older adults who were randomly selected from the resident registry of Kashiwa, Chiba, Japan.	People needing long-term care in the Japanese public insurance system, with cognitive impairment (MMSE score ≤18), a pacemaker, frailty, or sarcopenia at baseline.
Watanabe et al. 2017	People aged ≥65 years who were enrolled in the Obu Study of Health Promotion for the Elderly.	People with a history of Parkinson’s disease or stroke or with a MMSE score <18.
Weiss et al. 2021	Community-dwelling people aged ≥65 years who passed a brief cognitive screen	People with dysphagia
Yoshida et al. 2021	People who presented to the university for physical fitness assessment on June 22 and 23, 2019.	People with a history of cardiovascular disease and artificial joint replacement, and those whose systolic blood pressure exceeded 200 mmHg.
Yamanashi et al. 2018	People aged ≥60 years in Goto city, Nagasaki.	People with a history of stroke.

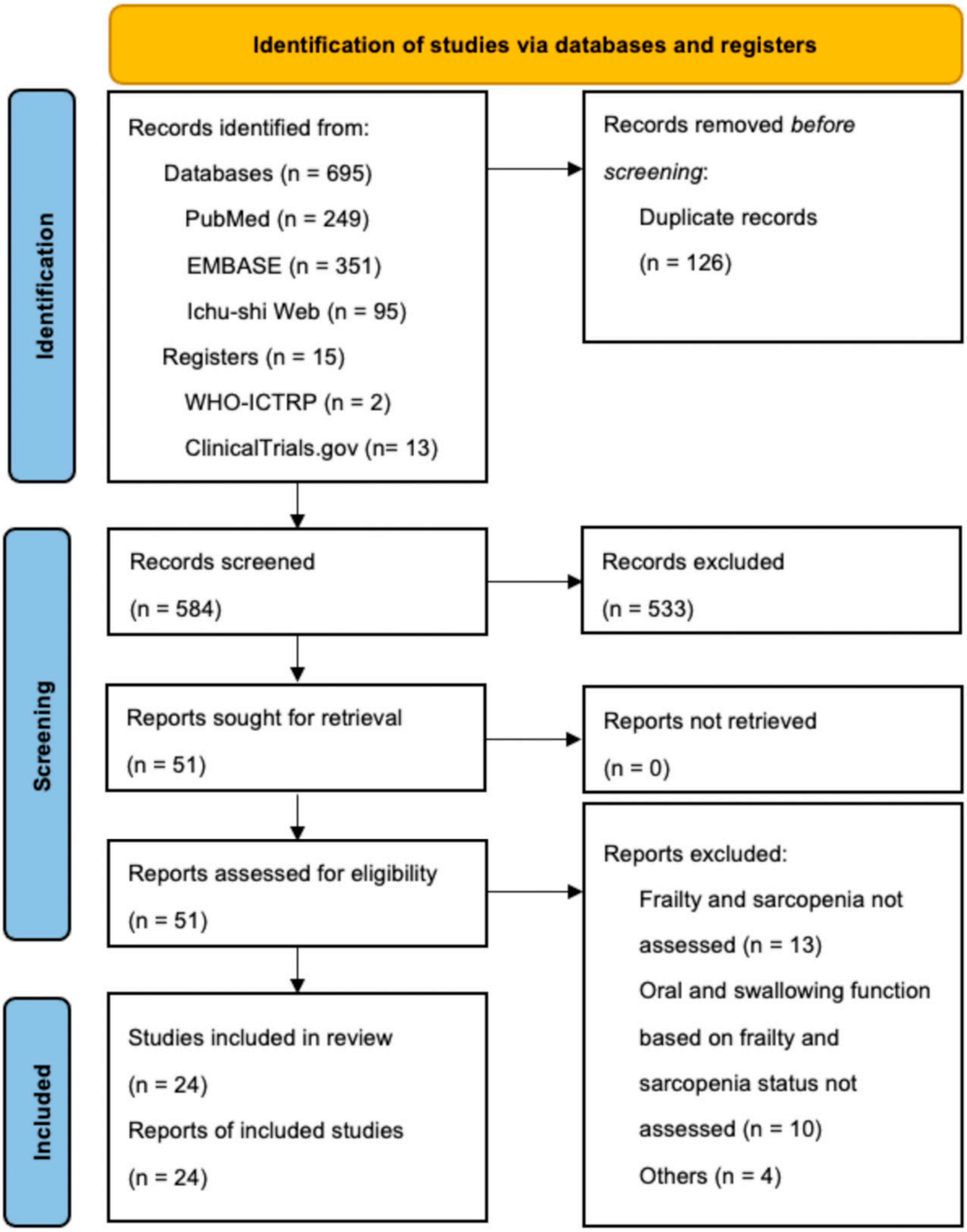

Figure S1. PRISMA 2020 flow diagram

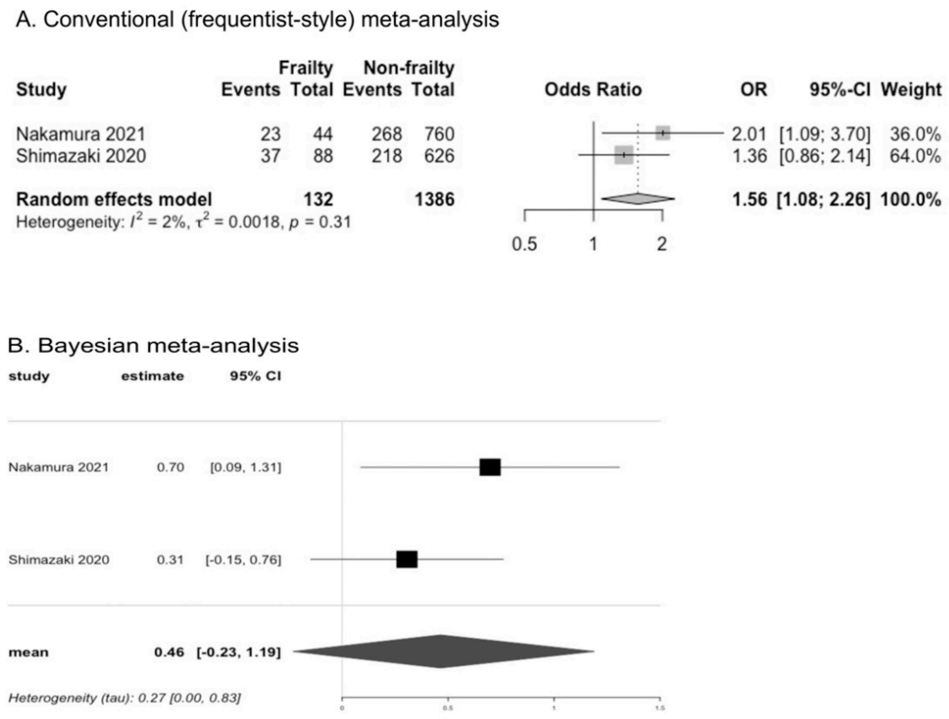

Figure S2. Comparison of low tongue pressure between individuals with and without frailty

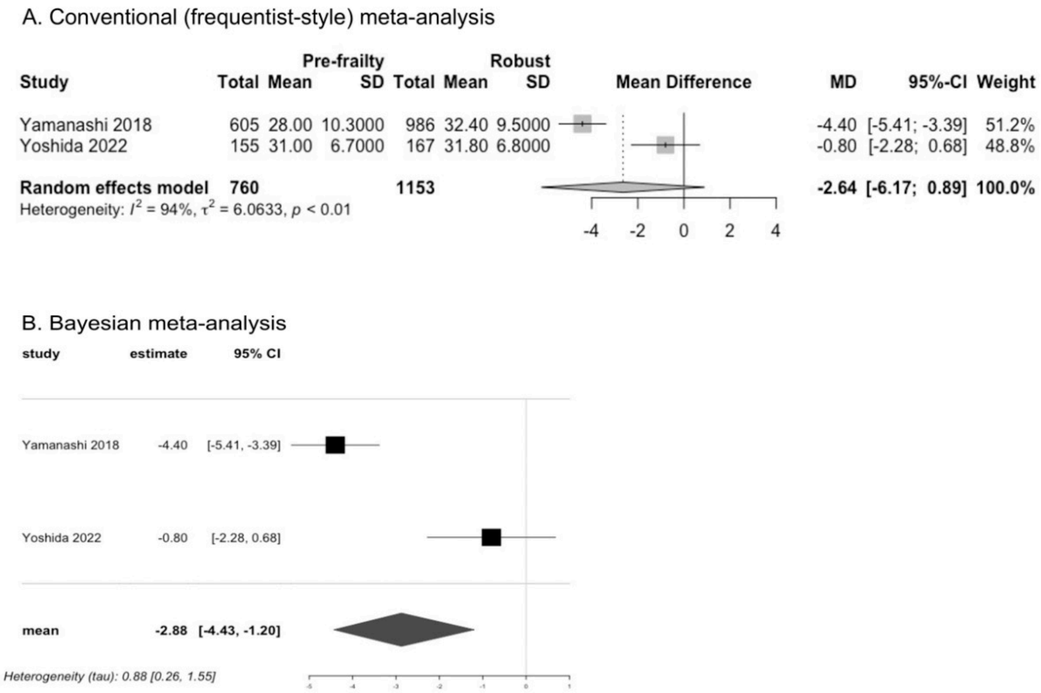

Figure S3. Comparison of tongue pressure between individuals with pre-frailty and robust individuals

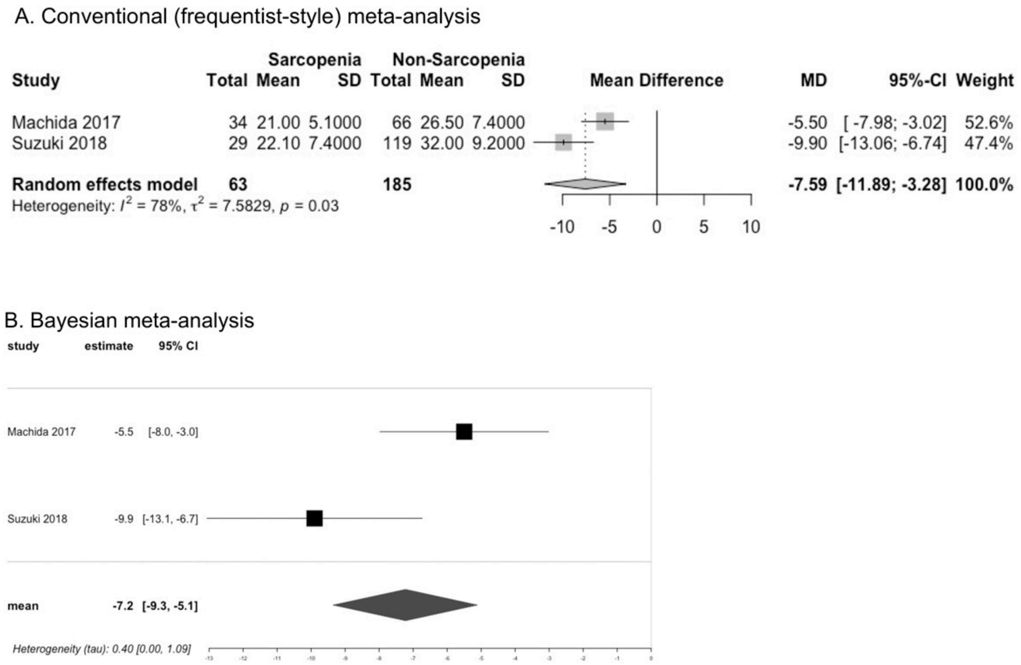

Figure S4. Comparison of tongue pressure between women with and without sarcopenia

A: Pa

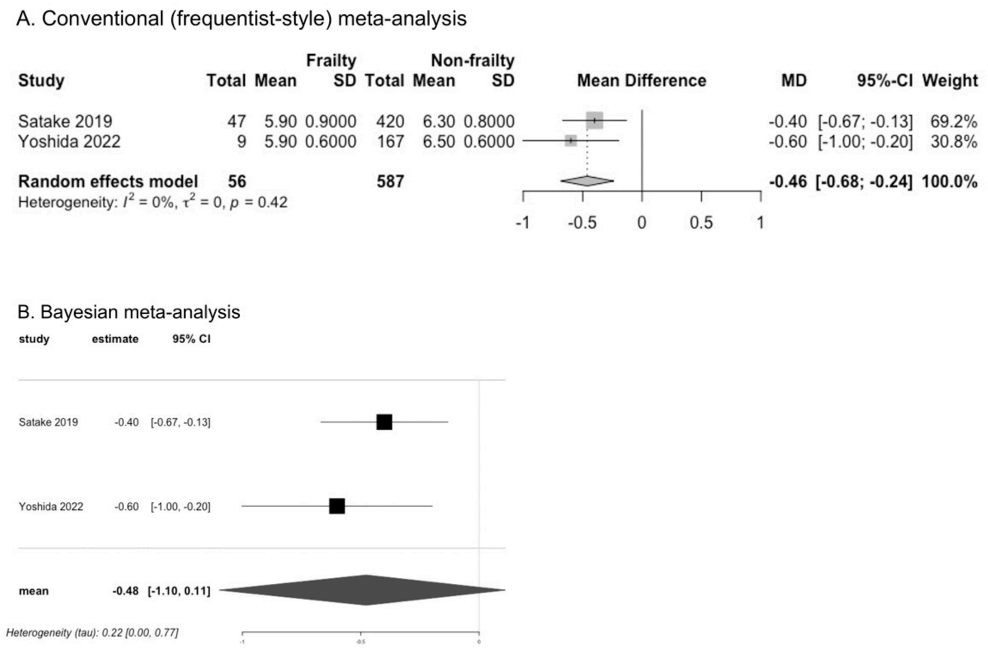
A. Conventional (frequentist-style) meta-analysis
Frailty
Non-frailty
Study Total Mean SD Total Mean SD Mean Difference MD 95%-CI Weight
Satake 2019 47 5.90 0.9000 420 6.30 0.8000 -0.40 [-0.67; -0.13] 69.2%
Yoshida 2022 9 5.90 0.6000 167 6.50 0.6000 -0.60 [-1.00; -0.20] 30.8%
Random effects model 56 587 -0.46 [-0.68; -0.24] 100.0%
Heterogeneity: I² = 0%, τ² = 0, p = 0.42
-1 -0.5 0 0.5 1
B. Bayesian meta-analysis
study estimate 95% CI
Satake 2019 -0.40 [-0.67, -0.13]
Yoshida 2022 -0.60 [-1.00, -0.20]
mean -0.48 [-1.10, 0.11]
Heterogeneity (tau): 0.22 [0.00, 0.77]

B: Ta

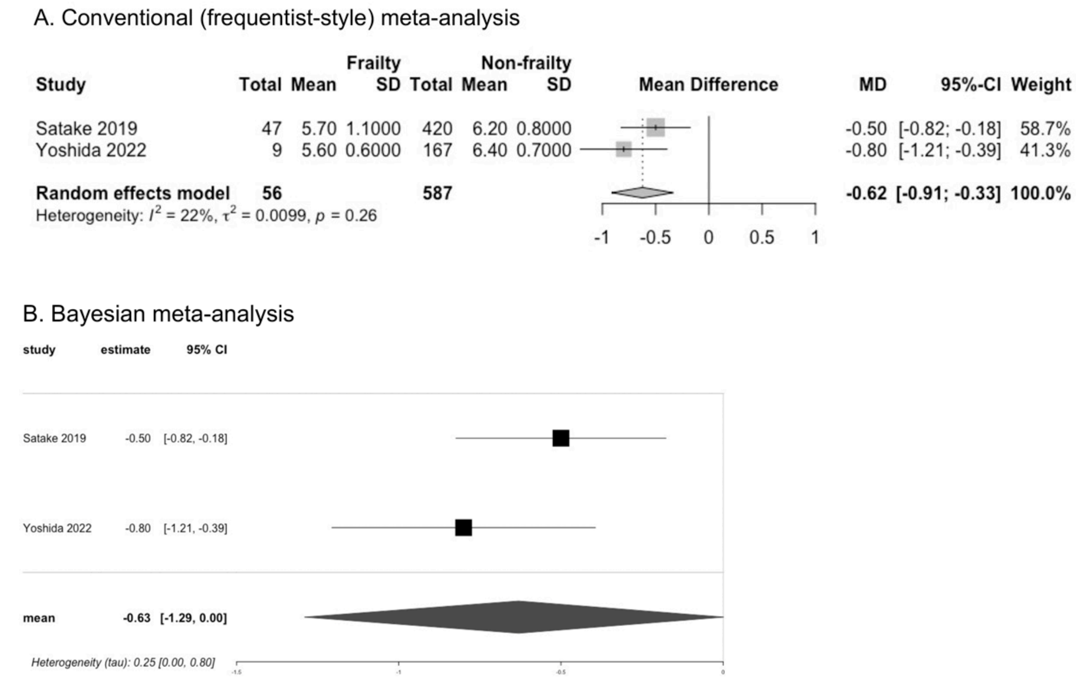
A. Conventional (frequentist-style) meta-analysis
Frailty
Non-frailty
Study Total Mean SD Total Mean SD Mean Difference MD 95%-CI Weight
Satake 2019 47 5.70 1.1000 420 6.20 0.8000 -0.50 [-0.82; -0.18] 58.7%
Yoshida 2022 9 5.60 0.6000 167 6.40 0.7000 -0.80 [-1.21; -0.39] 41.3%
Random effects model 56 587 -0.62 [-0.91; -0.33] 100.0%
Heterogeneity: I² = 22%, τ² = 0.0099, p = 0.26
-1 -0.5 0 0.5 1
B. Bayesian meta-analysis
study estimate 95% CI
Satake 2019 -0.50 [-0.82, -0.18]
Yoshida 2022 -0.80 [-1.21, -0.39]
mean -0.63 [-1.29, 0.00]
Heterogeneity (tau): 0.25 [0.00, 0.80]

C: Ka

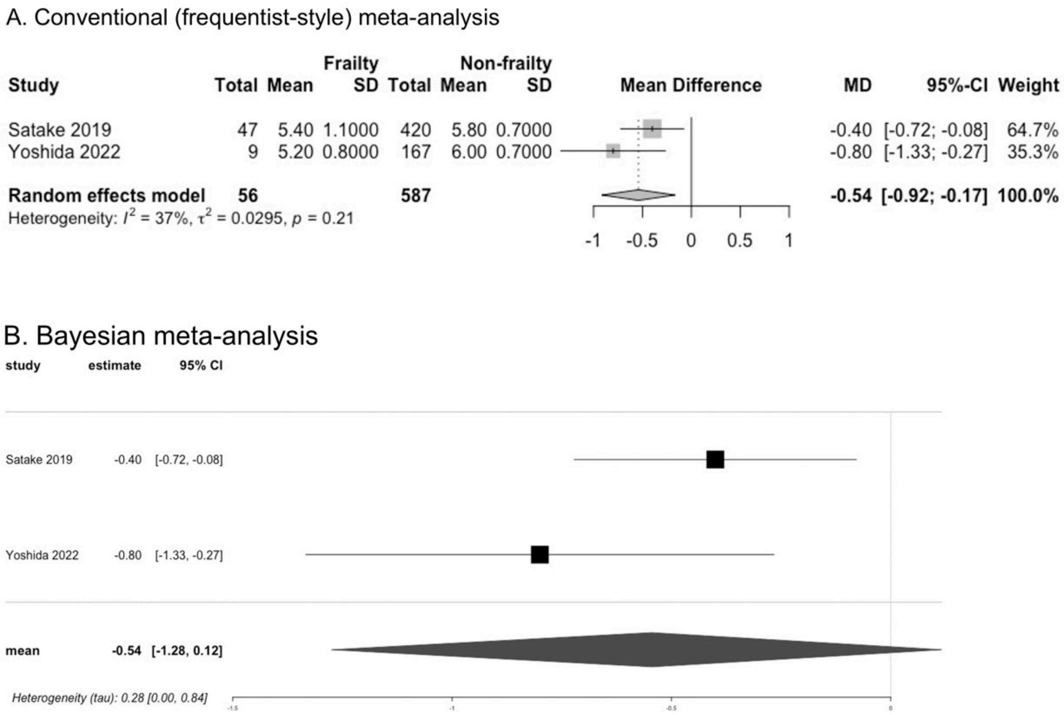

Figure S5. Comparison of the number of repetitions in oral diadochokinesis between individuals with and without frailty

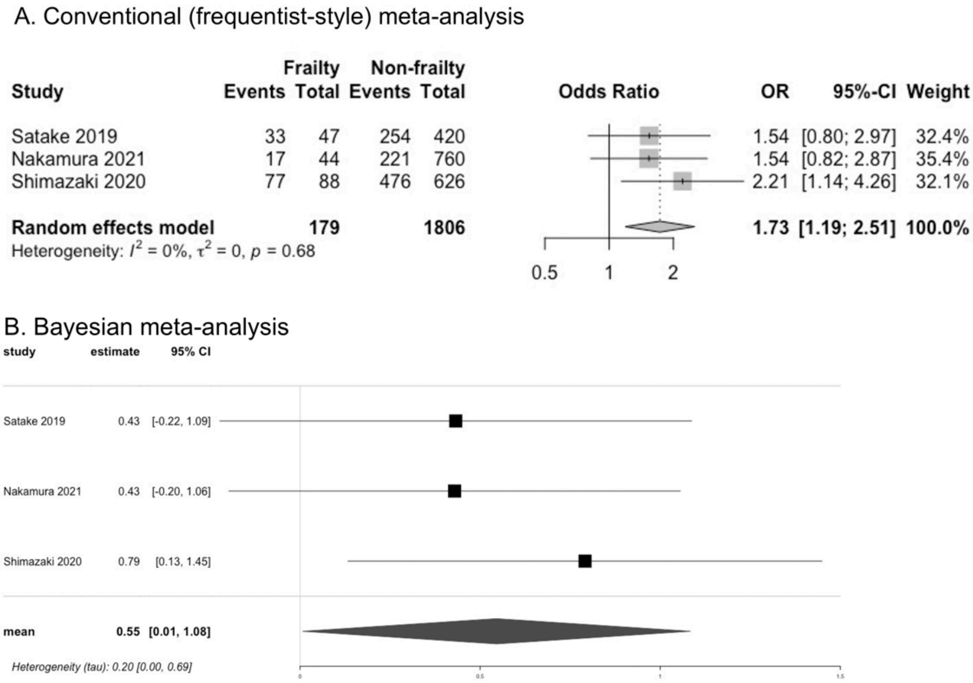

Figure S6. Comparison of low oral diadochokinesis in individuals with and without frailty

A: Pa

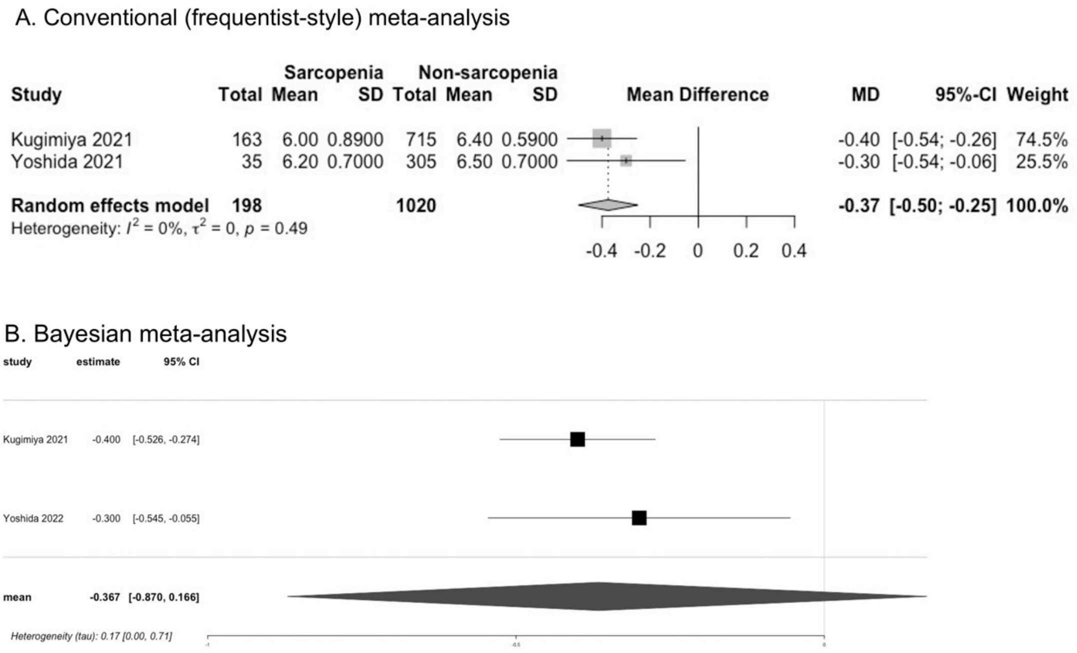
A. Conventional (frequentist-style) meta-analysis
Sarcopenia
Non-sarcopenia
Study
Total Mean SD Total Mean SD
Mean Difference
MD 95%-CI Weight
Kugimiya 2021 163 6.00 0.8900 715 6.40 0.5900 -0.40 [-0.54; -0.26] 74.5%
Yoshida 2021 35 6.20 0.7000 305 6.50 0.7000 -0.30 [-0.54; -0.06] 25.5%
Random effects model 198 1020 -0.37 [-0.50; -0.25] 100.0%
Heterogeneity: I^2 = 0%, τ^2 = 0, p = 0.49
-0.4 -0.2 0 0.2 0.4
B. Bayesian meta-analysis
study estimate 95% CI
Kugimiya 2021 -0.400 [-0.526, -0.274]
Yoshida 2022 -0.300 [-0.545, -0.055]
mean -0.367 [-0.870, 0.166]
Heterogeneity (tau): 0.17 [0.00, 0.71]

B：Ta

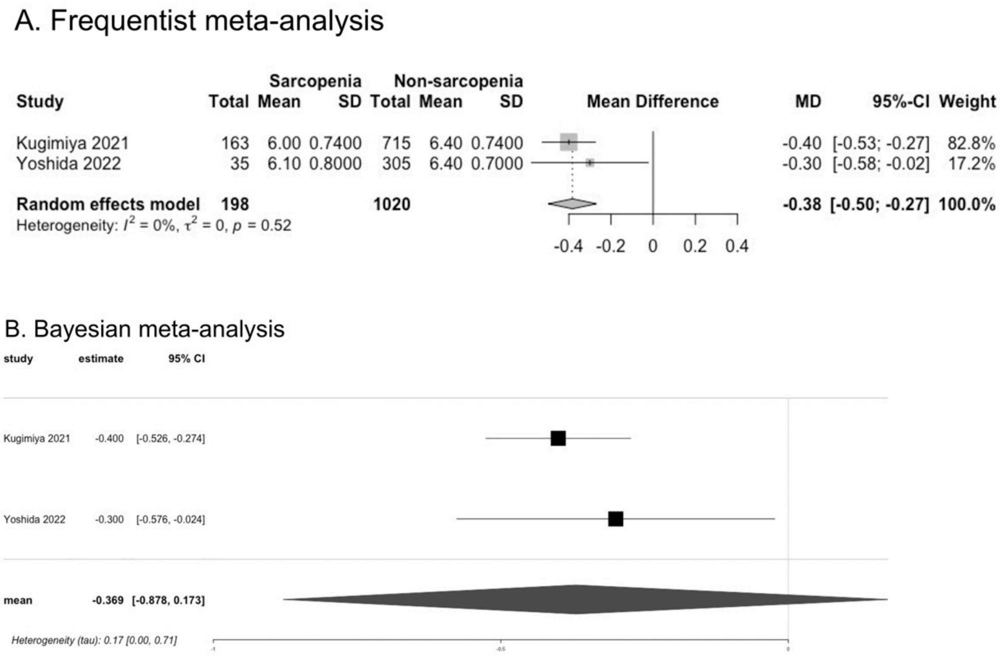
A. Frequentist meta-analysis
Sarcopenia
Non-sarcopenia
Study
Total Mean SD Total Mean SD
Mean Difference
MD 95%-CI Weight
Kugimiya 2021 163 6.00 0.7400 715 6.40 0.7400 -0.40 [-0.53; -0.27] 82.8%
Yoshida 2022 35 6.10 0.8000 305 6.40 0.7000 -0.30 [-0.58; -0.02] 17.2%
Random effects model 198 1020 -0.38 [-0.50; -0.27] 100.0%
Heterogeneity: I^2 = 0%, τ^2 = 0, p = 0.52
-0.4 -0.2 0 0.2 0.4
B. Bayesian meta-analysis
study estimate 95% CI
Kugimiya 2021 -0.400 [-0.526, -0.274]
Yoshida 2022 -0.300 [-0.576, -0.024]
mean -0.369 [-0.878, 0.173]
Heterogeneity (tau): 0.17 [0.00, 0.71]

C: Ka

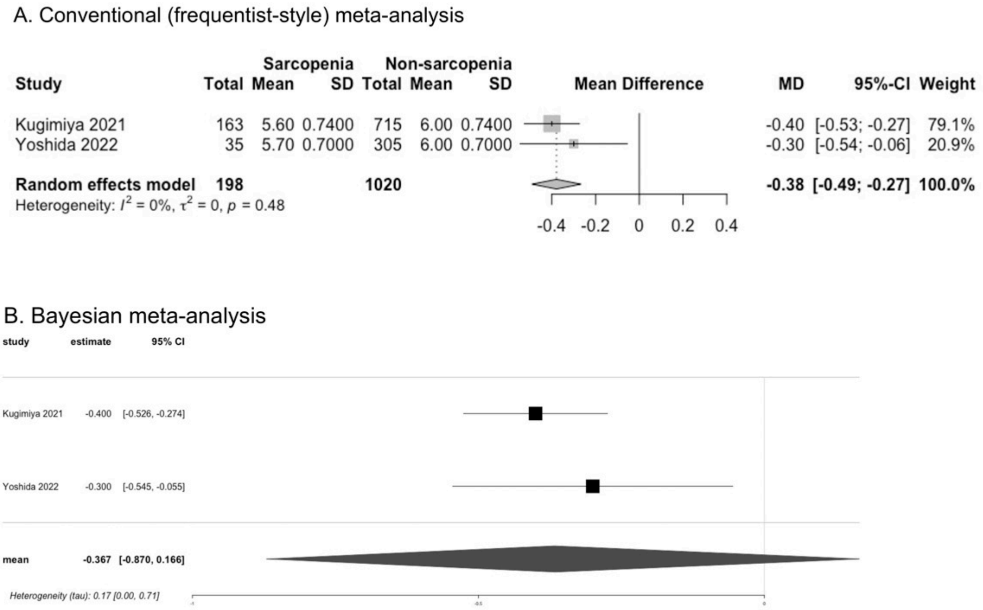

Figure S7. Comparison of number of repetitions in oral diadochokinesis between individuals with and without sarcopenia

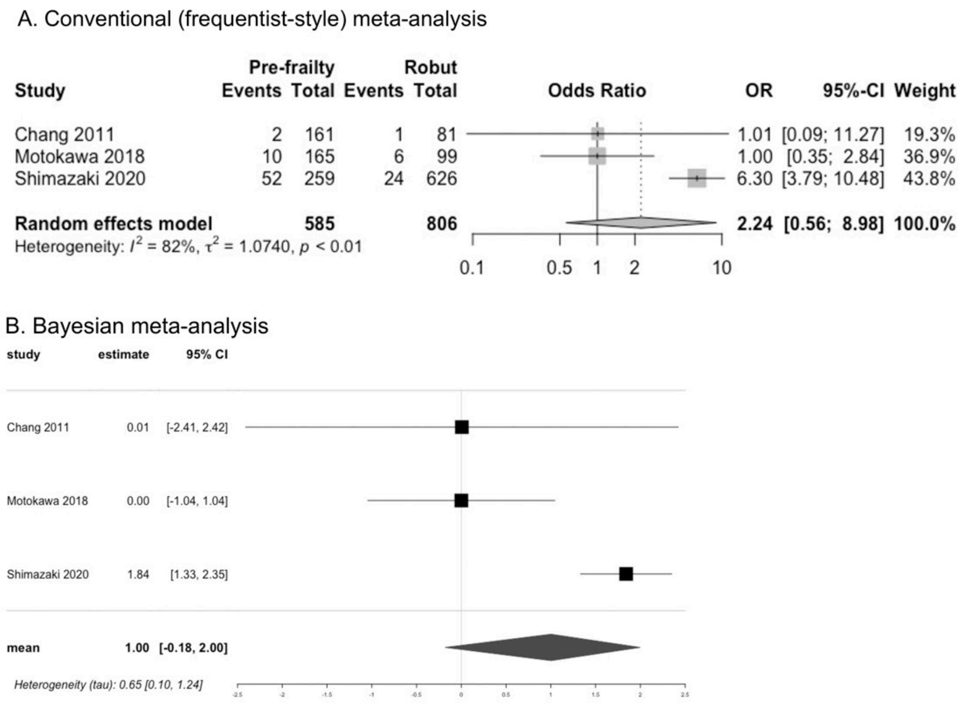

Figure S8. Comparison of dysphagia between individuals with pre-frailty and robust individuals

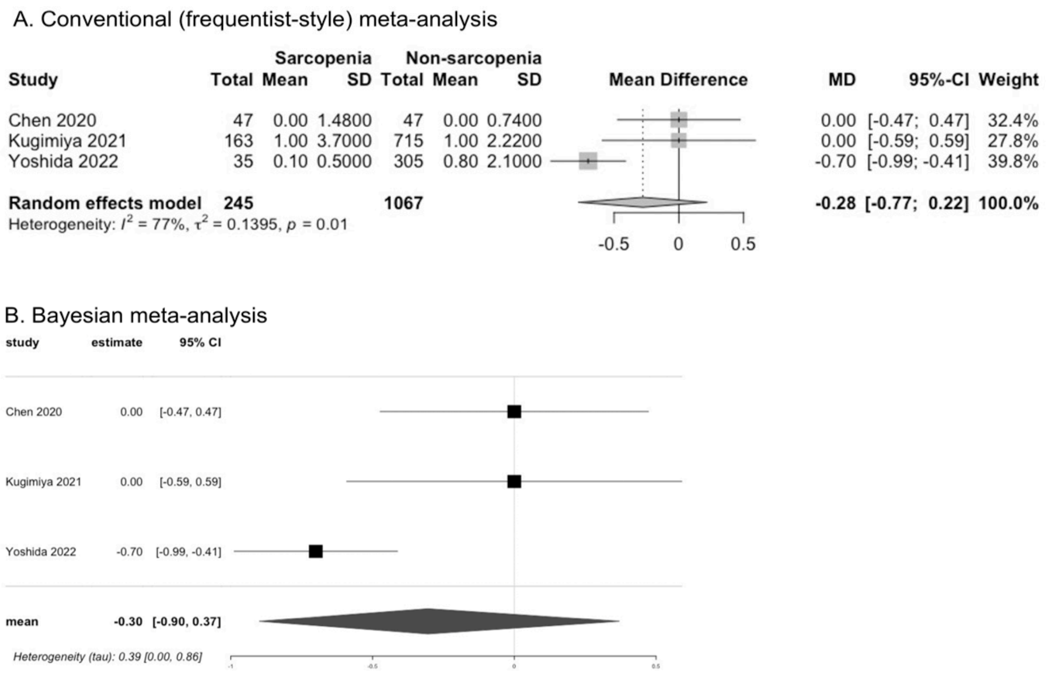

Figure S9. Comparison of Eating Assessment Tool-10 scores between individuals with and without sarcopenia.

References

44. Albani, V.; Nishio, K.; Ito, T.; Kotronia, E.; Moynihan, P.; Robinson, L.; Hanratty, B.; Kingston, A.; Abe, Y.; Takayama, M.; et al. Associations of poor oral health with frailty and physical functioning in the oldest old: results from two studies in England and Japan. *BMC Geriatr.* **2021**, *21*, 187.
45. Baba, H.; Watanabe, Y.; Miura, K.; Ozaki, K.; Matsushita, T.; Kondoh, M.; Okada, K.; Hasebe, A.; Ayabe, T.; Nakamura, K.; et al. Oral frailty and carriage of oral Candida in community-dwelling older adults (Check-up to discover Health with Energy for senior Residents in Iwamizawa; CHEER Iwamizawa). *Gerodontology* **2022**, *39*, 49–58.
46. Bahat, G.; Yilmaz, O.; Durmazoglu, S.; Kilic, C.; Tascioglu, C.; Karan, M.A. Association between Dysphagia and Frailty in Community Dwelling Older Adults. *J. Nutr. Health Aging* **2019**, *23*, 571–577.
47. Buehring, B.; Hind, J.; Fidler, E.; Krueger, D.; Binkley, N.; Robbins, J. Tongue strength is associated with jumping mechanography performance and handgrip strength but not with classic functional tests in older adults. *J. Am. Geriatr. Soc.* **2013**, *61*, 418–422.
48. Chang, K.V.; Wu, W.T.; Chen, L.R.; Wang, H.I.; Wang, T.G.; Han, D.S. Suboptimal Tongue Pressure Is Associated with Risk of Malnutrition in Community-Dwelling Older Individuals. *Nutrients* **2021**, *13,* 1821.
49. Chaleekrua, S.; Janpol, K.; Wattanapan, P. Swallowing Problems among Community-Dwelling Elderly in Northeastern Thailand. *J. Prim. Care Community Health* **2021**, *12*, 21501327211019596.
50. González-Fernández, M.; Humbert, I.; Winegrad, H.; Cappola, A.R.; Fried, L.P. Dysphagia in old-old women: prevalence as determined according to self-report and the 3-ounce water swallowing test. *J. Am. Geriatr. Soc.* **2014**, *62*, 716–720.
51. Hironaka, S.; Kugimiya, Y.; Watanabe, Y.; Motokawa, K.; Hirano, H.; Kawai, H.; Kera, T.; Kojima, M.; Fujiwara, Y.; Ihara, K.; et al. Association between oral, social, and physical frailty in community-dwelling older adults. *Arch. Gerontol. Geriatr.* **2020**, *89*, 104105.
52. Hoshino, D.; Hirano, H.; Edahiro, A.; Motokawa, K.; Shirobe, M.; Watanabe, Y.; Motohashi, Y.; Ohara, Y.; Iwasaki, M.; Maruoka, Y.; et al. Association between Oral Frailty and Dietary Variety among Community-Dwelling Older Persons: A Cross-Sectional Study. *J. Nutr. Health Aging* **2021**, *25*, 361–368.
53. Iwasaki, M.; Motokawa, K.; Watanabe, Y.; Shirobe, M.; Inagaki, H.; Edahiro, A.; Ohara, Y.; Hirano, H.; Shinkai, S.; Awata, S. Association between Oral Frailty and Nutritional Status among Community-Dwelling Older Adults: the Takashimadaira Study. *J. Nutr. Health Aging* **2020**, *24*, 1003–1010.
54. Iwasaki, M.; Motokawa, K.; Watanabe, Y.; Shirobe, M.; Inagaki, H.; Edahiro, A.; Ohara, Y.; Hirano, H.; Shinkai, S.; Awata, S. A Two-Year Longitudinal Study of the Association between Oral Frailty and Deteriorating Nutritional Status among Community-Dwelling Older Adults. *Int. J. Environ. Res. Public Health* **2020**, *18,* 213.
55. Iwasaki, M.; Watanabe, Y.; Motokawa, K.; Shirobe, M.; Inagaki, H.; Motohashi, Y.; Mikami, Y.; Taniguchi, Y.; Osuka, Y.; Seino, S.; et al. Oral frailty and gait performance in community-dwelling older adults: findings from the Takashimadaira study. *J. Prosthodont. Res.* **2021**, *65*, 467–473.
56. Kaji, A.; Hashimoto, Y.; Kobayashi, Y.; Sakai, R.; Okamura, T.; Miki, A.; Hamaguchi, M.; Kuwahata, M.; Yamazaki, M.; Fukui, M. Sarcopenia is associated with tongue pressure in older patients with type 2 diabetes: A cross-sectional study of the KAMOGAWA-DM cohort study. *Geriatr. Gerontol. Int.* **2019**, *19*, 153–158.
57. Kugimiya, Y.; Motokawa, K.; Yamamoto, K.; Hayakawa, M.; Mikami, Y.; Iwasaki, M.; Ohara, Y.; Shirobe, M.; Edahiro, A.; Watanabe, Y.; et al. Relationship between the rate of a decreased oral function and the nutrient intake in community-dwelling older persons: An examination using oral function-related items in a questionnaire for latter-stage elderly people. *Nihon Ronen Igakkai Zasshi* **2021**, *58*, 91–100.
58. Koyama, S.; Otobe, Y.; Suzuki, M.; Kimura, Y.; Ishiyama, D.; Kojima, I.; Masuda, H.; Kusumi, H.; Yamada, M. Relationship between the Kihon Checklist and all-cause hospitalization among community-dwelling older adults. *Geriatr. Gerontol. Int.* **2022**, *22*, 132–137.
59. Molfenter, S.M.; Lenell, C.; Lazarus, C.L. Volumetric Changes to the Pharynx in Healthy Aging: Consequence for Pharyngeal Swallow Mechanics and Function. *Dysphagia* **2019**, *34*, 129–137.
60. Morishita, M.; Ikeda, T.; Saito, N.; Sanou, M.; Yasuda, M.; Takao, S. Relationship between oral function and life-space mobility or social networks in community-dwelling older people: A cross-sectional study. *Clin. Exp. Dent. Res.* **2021**, *7*, 552–560.
61. Murotani, Y.; Hatta, K.; Takahashi, T.; Gondo, Y.; Kamide, K.; Kabayama, M.; Masui, Y.; Ishizaki, T.; Matsuda, K.I.; Mihara, Y.; et al. Oral Functions Are Associated with Muscle Strength and Physical Performance in Old-Old Japanese. *Int. J. Environ. Res. Public Health* **2021**, *18,* 13199.
62. Nakao, Y.; Yamashita, T.; Honda, K.; Katsuura, T.; Hama, Y.; Nakamura, Y.; Ando, K.; Ishikura, R.; Kodama, N.; Uchiyama, Y.; et al. Association Among Age-Related Tongue Muscle Abnormality, Tongue Pressure, and Presbyphagia: A 3D MRI Study. *Dysphagia* **2021**, *36*, 483–491.
63. Nishida, T.; Yamabe, K.; Honda, S. Dysphagia is associated with oral, physical, cognitive and psychological frailty in Japanese community-dwelling elderly persons. *Gerodontology* **2020**, *37*, 185–190.

64. Nishimoto, M.; Tanaka, T.; Takahashi, K.; Unyaporn, S.; Fujisaki-Sueda-Sakai, M.; Yoshizawa, Y.; Iijima, K. Oral frailty is associated with food satisfaction in community-dwelling older adults. *Nihon Ronen Igakkai Zasshi* **2020**, *57*, 273–281.
65. Iwai-Saito, K.; Shobugawa, Y.; Aida, J.; Kondo, K. Frailty is associated with susceptibility and severity of pneumonia in older adults (A JAGES multilevel cross-sectional study). *Sci. Rep.* **2021**, *11*, 7966.
66. Suzuki, H.; Ayukawa, Y.; Ueno, Y.; Atsuta, I.; Jinnouchi, A.; Koyano, K. Relationship between Maximum Tongue Pressure Value and Age, Occlusal Status, or Body Mass Index among the Community-Dwelling Elderly. *Medicina* **2020**, *56*, 623.
67. Takeuchi, N.; Sawada, N.; Ekuni, D.; Morita, M. Oral diadochokinesis is related to decline in swallowing function among community-dwelling Japanese elderly: a cross-sectional study. *Aging Clin. Exp. Res.* **2021**, *33*, 399–405.
68. Takehara, S.; Hirani, V.; Wright, F.A.C.; Naganathan, V.; Blyth, F.M.; Le Couteur, D.G.; Waite, L.M.; Seibel, M.J.; Handelsman, D.J.; Cumming, R.G. Appetite, oral health and weight loss in community-dwelling older men: an observational study from the Concord Health and Ageing in Men Project (CHAMP). *BMC Geriatr.* **2021**, *21*, 255.
69. Tanaka, T.; Hirano, H.; Ohara, Y.; Nishimoto, M.; Iijima, K. Oral Frailty Index-8 in the risk assessment of new-onset oral frailty and functional disability among community-dwelling older adults. *Arch. Gerontol. Geriatr.* **2021**, *94*, 104340.
70. Yamada, Y.; Nanri, H.; Watanabe, Y.; Yoshida, T.; Yokoyama, K.; Itoi, A.; Date, H.; Yamaguchi, M.; Miyake, M.; Yamagata, E.; et al. Prevalence of Frailty Assessed by Fried and Kihon Checklist Indexes in a Prospective Cohort Study: Design and Demographics of the Kyoto-Kameoka Longitudinal Study. *J. Am. Med. Dir. Assoc.* **2017**, *18*, e733–e737.
